# RAFT polymer cross-coupling with boronic acids†
†Electronic supplementary information (ESI) available: Experimental details, NMR spectra of all new compounds, GPC traces, MALDI-MS. See DOI: 10.1039/c8sc01862f


**DOI:** 10.1039/c8sc01862f

**Published:** 2018-07-18

**Authors:** Hartwig Golf, Riley O'Shea, Carl Braybrook, Oliver Hutt, David W. Lupton, Joel F. Hooper

**Affiliations:** a School of Chemistry , Monash University , Clayton , Melbourne , VIC 3800 , Australia . Email: david.lupton@monash.edu ; Email: joel.hooper@monash.edu; b CSIRO , Research Way , Melbourne , VIC 3168 , Australia

## Abstract

The ability to modify the thiocarbonylthio end-groups of RAFT polymers is important for applications where an inert or highly functionalised material is required. Here we report a copper promoted cross-coupling reaction between RAFT polymer end-groups and aryl boronic acids. This method gives high conversion to the modified polymers, and is compatible with a wide variety of functional molecules.

## Abstract 2

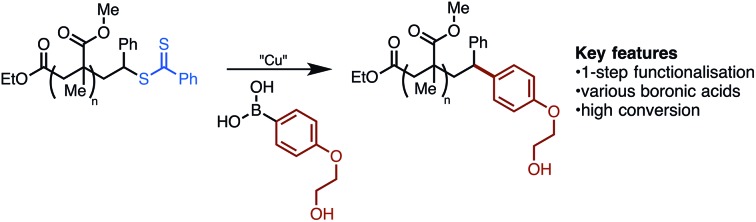
A one step modification of RAFT polymers to give functionalised materials.

## Introduction

**Scheme 1:**
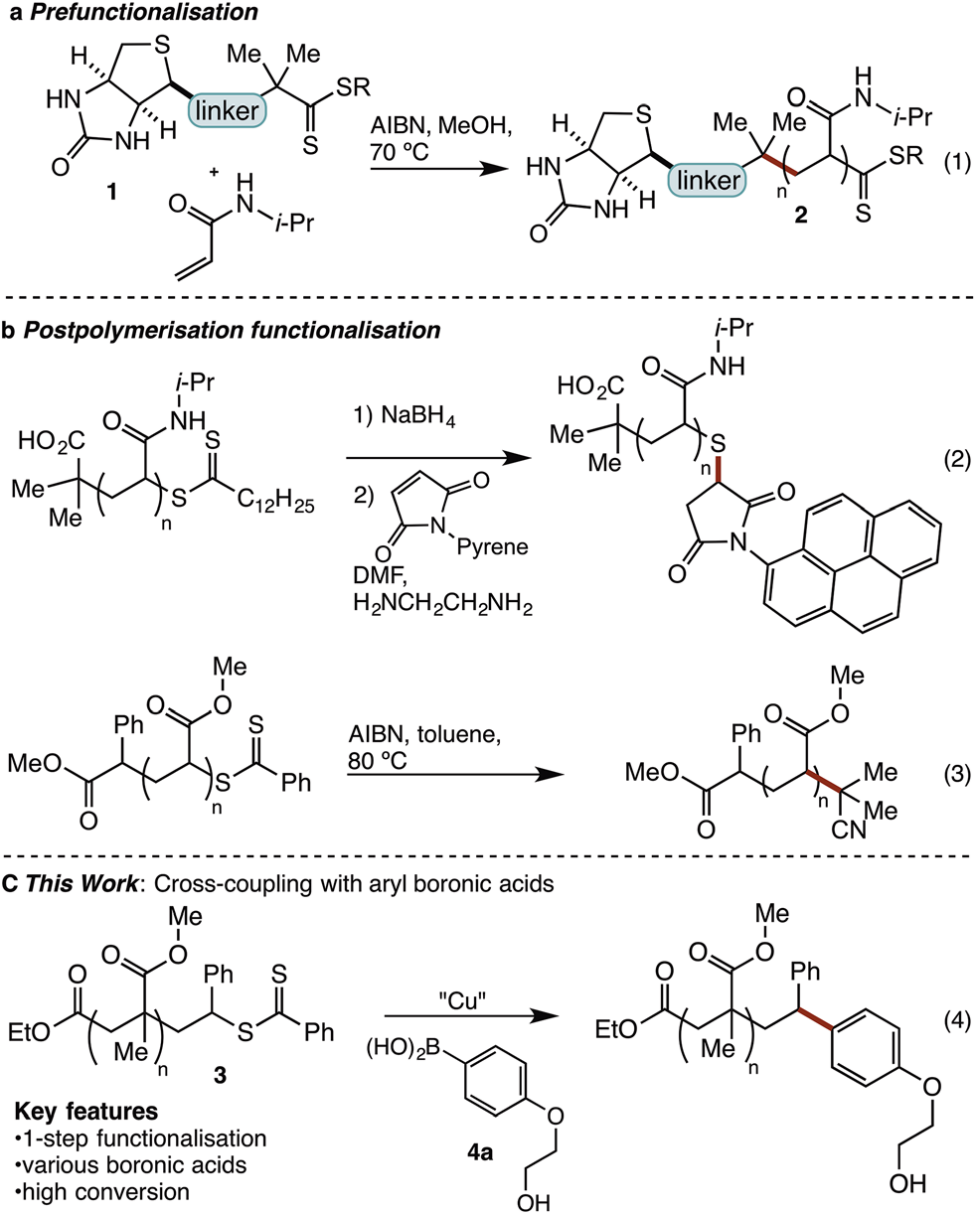
Existing approaches to RAFT end-group removal.

Reversible addition fragmentation chain transfer (RAFT) polymerisation is a powerful method for the synthesis of narrow polydispersity polymers.^1^ Applicable to most monomers, RAFT is increasingly being deployed in sophisticated applications, such as in biomedical science.^2^–^7^ Central to such applications is the ability to prepare robust narrow dispersity polymer conjugates. For example, in 2006 Hong reported formation of polyacrylamide biotin conjugate **2** using prefunctionalised RAFT agent **1** (eqn (1)).6a,^8^ While this approach provided a biotin polyacrylamide conjugate it retained the thiocarbonylthio end-group, which is generally considered undesirable as it can lead to instability, discolouration, or unpleasant odours.^9^ An alternate strategy to the formation of polymer conjugates involves post-polymerisation functionalization (Scheme 1b). This often involves a two-step cleavage of the thiocarbonyl group to leave a free thiol,^10^ and coupling *via* thiol ene or other sulfur specific conjugations (eqn (2)).^11^ This common strategy suffers from its multistep nature while leaving a sulphur-containing tether, which may be prone to oxidation, elimination or exchange reactions.^12^ A number of functionalisations allow removal of the sulfur-containing end-group.^13^ In early studies, thermal eliminations were developed to generate alkene terminated polymers,^14^ while more recently re-initiation and quenching with a hydrogen atom source^15^ or alkyl radical has been developed (eqn (3)).13b,^16^ Recently reported desulfurisation to generate bromine terminated polymers has been described by Lunn using a 2-step aminolysis/bromination,^17^ while Armes used oxidative methods to give hydroxyl end-groups.^18^ In addition, Lunn and Sumerlin have independently exploited photoactivation to deliver hydrogen terminated polymers.^19^ Although existing techniques to remove sulfur have strengths, they often require multiple reactions and are only capable of introducing simple functionality.

Despite recent advances in the use of transition metals for the activation and functionalisation of C–S bonds,^20^ we noted with interest that this strategy has not been applied to RAFT endgroups. In principle, such a strategy should allow deletion of the sulfur end-group and introduction of new functionality *via* a robust C–C bond. Herein, we report studies on this topic that have led to the discovery of a Cu[ii] promoted coupling of RAFT polymers **3** with aryl boronic acid **4** (eqn (4)). This method exploits readily available boronic acids, deletes the thiocarbonylthio group, and introduces the conjugate with a stable C–C bond.

## Results and discussion

Studies began by examining the cross-coupling of small molecule polystyrene surrogates (*i.e.***5**) with aryl boronic acid (**4b**) (Table 1). Pleasingly a range of Cu[0] and [ii] complexes gave the desired diarylmethane **6**, albeit in low yield (Table 1, entries 1–5). Cu(BF_4_)_2_·H_2_O proved the best copper promoter, giving the product in 35% isolated yield (Table 1, entry 5). Solvent screening identified chlorinated solvents, and particularly 1,2-dichloroethane, as suitable for this reaction lifting the yield to 54% (Table 1, entries 6–10). This outcome was improved with the simpler phenyl-thiocarbonylthio substrate (**5b**) (Table 1, entry 11). Finally, through attempts to dry the Cu(BF_4_)_2_·H_2_O salt over MgSO_4_ in EtOAc, an amorphous Cu(BF_4_)_2_·H_2_O·EtOAc adduct formed that proved more active than the Cu(BF_4_)_2_·H_2_O salt, leading to the isolation of diarylmethane **6** in 85% yield from the coupling of phenyl-thiocarbonylthio **5b** with boronic acid **4b**.

**Table 1:** Optimisation of the cross-coupling of thiocarbonylthio **5** with boronic acid **4b**

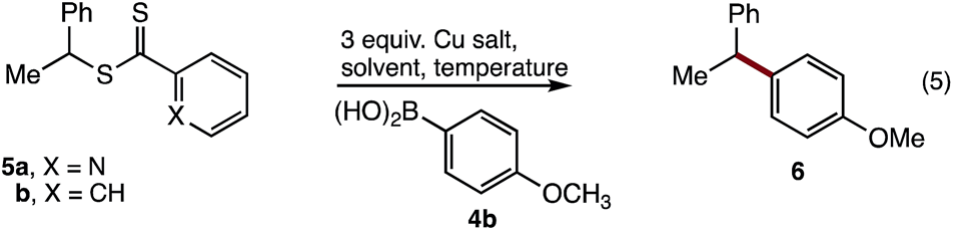					
Entry **5**		Cu salt	Solvent	Temp	Yield^*a*^
1	**a**	Cu(acac)	THF	66	0
2	′′	CuCl_2_·H_2_O	′′	′′	Trace
3	′′	Cu powder	′′	′′	12
4	′′	Cu(OAc)_2_·H_2_O	′′	′′	21
5	′′	Cu(BF_4_)_2_·H_2_O	′′	′′	35
6	′′	′′	Dioxane	′′	12
7	′′	′′	DMF	′′	21
8	′′	′′	Toluene	′′	16
9	′′	′′	CH_2_Cl_2_	40	42
10	′′	′′	DCE	66	54
11^*a*^	**b**	′′	DCE	80	67
12^*a*^	**b**	Cu(BF_4_)_2_·H_2_O·EtOAc	′′	80	85

^*a*^Isolated yields.

**Fig. 1:**
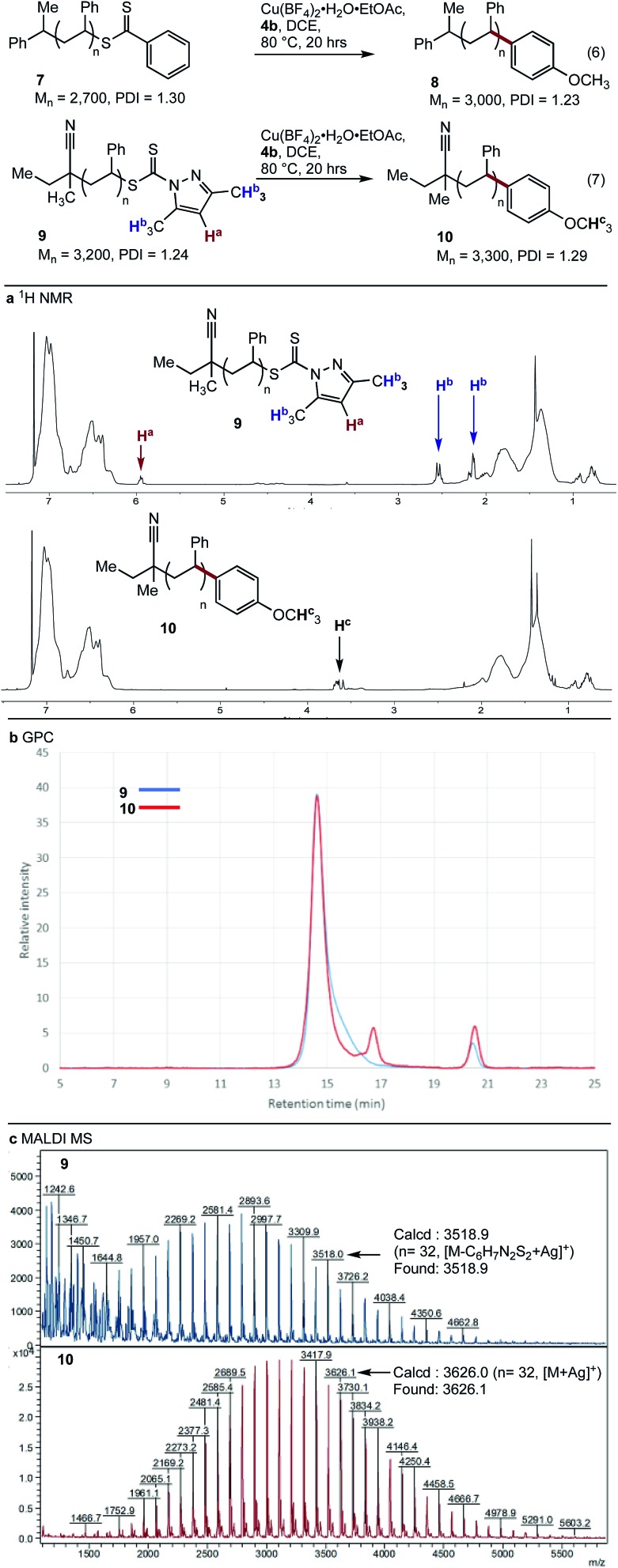
Cross-coupling of polystyrenes **7** and **9** with boronic acid **4b.**

With conditions optimised for end-group modification of a polystyrene surrogate, the cross-coupling with a low molecular weight polystyrene bearing a phenyl-thiocarbonylthio end-group (*i.e.***7**) was examined (Fig. 1). Pleasingly, the conditions were well suited to polymer **7** and gave the polymer conjugate **8** with >95% end-group conversion, as judged by ^1^H NMR spectroscopy (see ESI†). The conditions were equally suited (>95% conversion) to the cross-coupling of polystyrene bearing the recently reported dimethylpyrazole end-group (*i.e.***9**) with aryl boronic acid **4b** (eqn (7)).^21^ The ^1^H NMR spectra of the resultant conjugate **10** showed clear disappearance of signals assigned to the pyrazole end-group (*i.e.***H^a^** and **H^b^**). In addition, a broad signal at ∼3.75 ppm was seen, consistent with the methoxy group from the aryl boronic acid (Fig. 1a).

As expected, analysis of polymers **9** and **10** by gel permeation chromatography (GPC) showed very little change in the molecular weight or dispersity (Fig. 1b). MALDI-MS analysis of polymers **9** and **10** clearly showed incorporation of the aryl end-group (Fig. 1c). Finally, analysis of polymer **10** by ICP-MS after only simple purification by precipitation showed a residual copper content of <10 ppm.

**Fig. 2:**
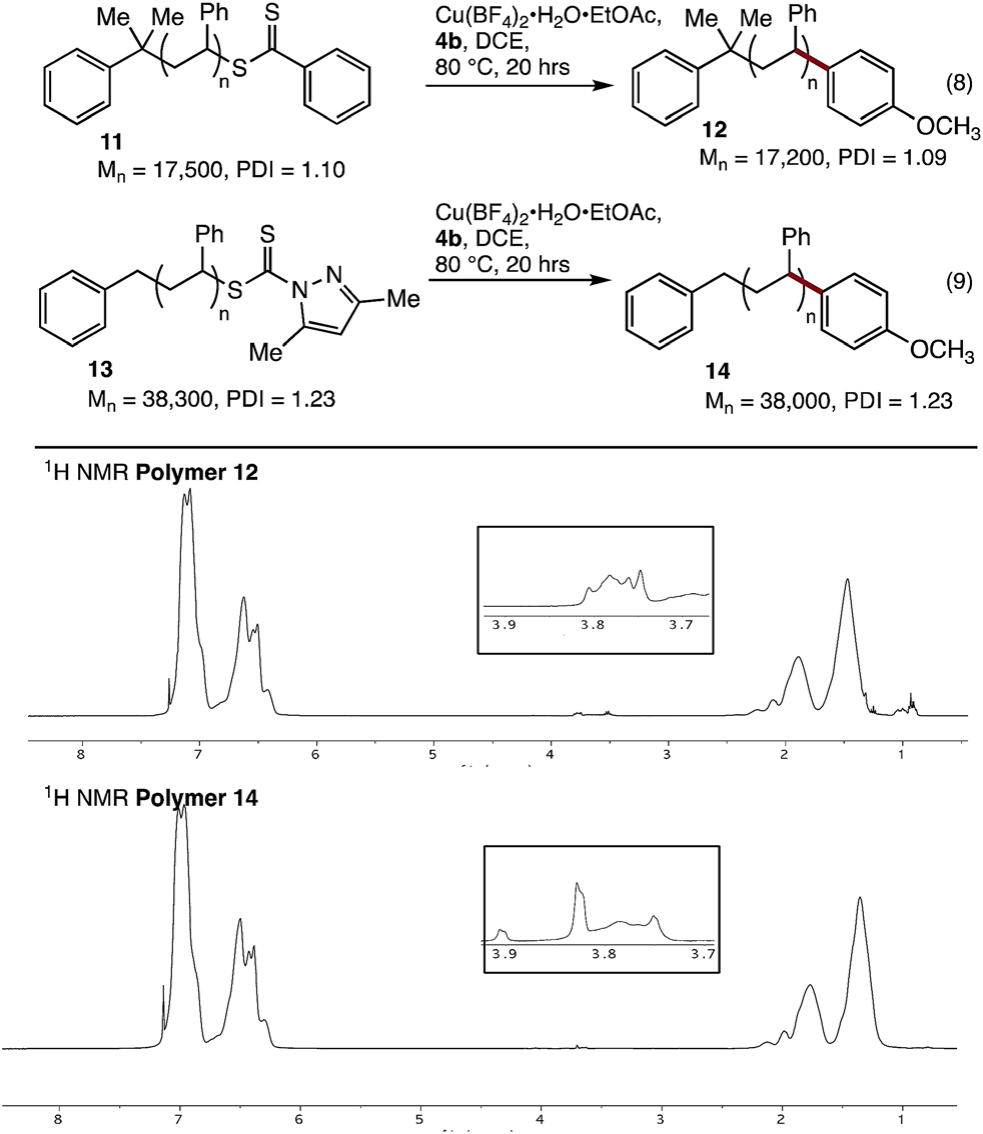
Cross-coupling of polystyrenes **11** and **13** with boronic acid **4b**.

We next tested our coupling conditions on higher molecular weight polystyrenes **11** and **13** (Fig. 2). Coupling under our standard conditions, with 3 equivalents of Cu relative to the endgroup, resulted in incomplete conversion. However, when the equivalents of Cu and **4b** were increased so as to maintain the same concentration as in the previous experiments (∼50 mg mL^–1^ Cu(BF_4_)_2_·H_2_O·EtOAc, 16 equiv for **11**, 36 equiv. for **13**), clear incorporation of the new methoxyphenyl end-group could be observed. While quantitative end-group analysis by ^1^H NMR spectroscopy is difficult at these higher molecular weights, these results clearly show that our coupling conditions are viable with these substrates, as long as the concentration of reagents is maintained at the optimised level.

**Scheme 2:**
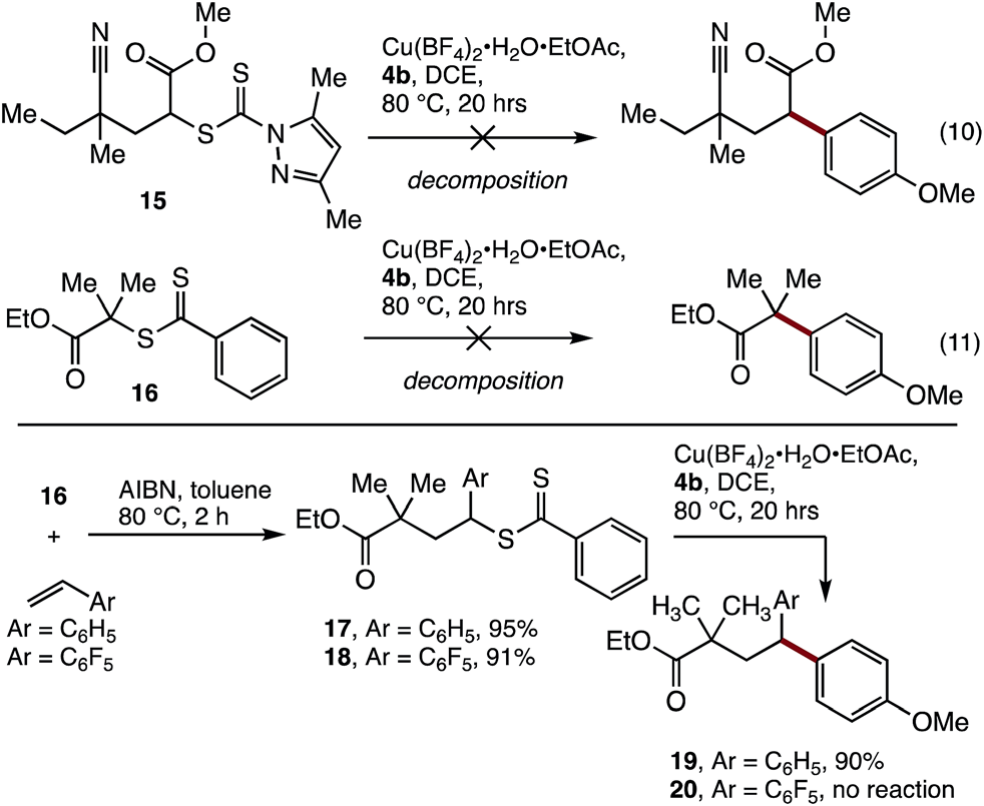
SUMI/coupling strategy for the modification of methacrylates.

Having established cross-coupling with polystyrene, the applicability of these conditions to the coupling of model polyacrylate (**15**) and polymethacrylate (**16**) small molecules with aryl boronic acid **4b** was examined. Unfortunately, these reactions resulted in decomposition of the starting materials (Scheme 2), indicating that benzylic activation of the reactive site is required for effective coupling.

The requirement for benzylic activation at the reactive site is consistent with previous Cu-promoted activation of C_sp^3^_–S bonds,^22^ where benzylic activation is thought to stabilise a proposed carbocation intermediate. In addition, a radical-based mechanism is also possible, where copper coordination to the dithiocarbonyl group is followed by C–S bond clevage to produce a benzylic radical and concomitant single-electron oxidation of Cu. It has been previously shown that Cu[0]^23^ or Fe[0]^24^ can be used to initiate RAFT polymerisations, suggesting that C_sp^3^_ radicals can be generated under these conditions.

To address this limitation, we examined the incorporation of styrene units to the end of a polymethacrylate to modify its reactivity. The synthesis of a block co-polymer was considered, although this would complicate the characterisation of the polymer by MALDI-MS, and would limit the utility of the material produced. As an alternative, a single unit monomer insertion (SUMI)^25^ strategy was examined, where a single styrene unit may be inserted into the end of the polymer. This strategy would provide the required reactivity for functionalization and deliver a well-defined material for analysis, while having minimal impact on the bulk properties of the polymethacrylate.

Thus, treatment of the model substrate **16** with AIBN and two equivalents of styrene gave the SUMI surrogate **17** in excellent yield (Scheme 2). The incorporation of pentafluorostyrene was also successful (**18**), which was included to probe the effect of increased cation/radical stabilisation at the benzylic position. While **18** proved to be unreactive under the cross-coupling conditions, the styrene insertion product **17** underwent efficient coupling with boronic acid **4b** to deliver **19** in 90% yield.^26^

**Fig. 3:**
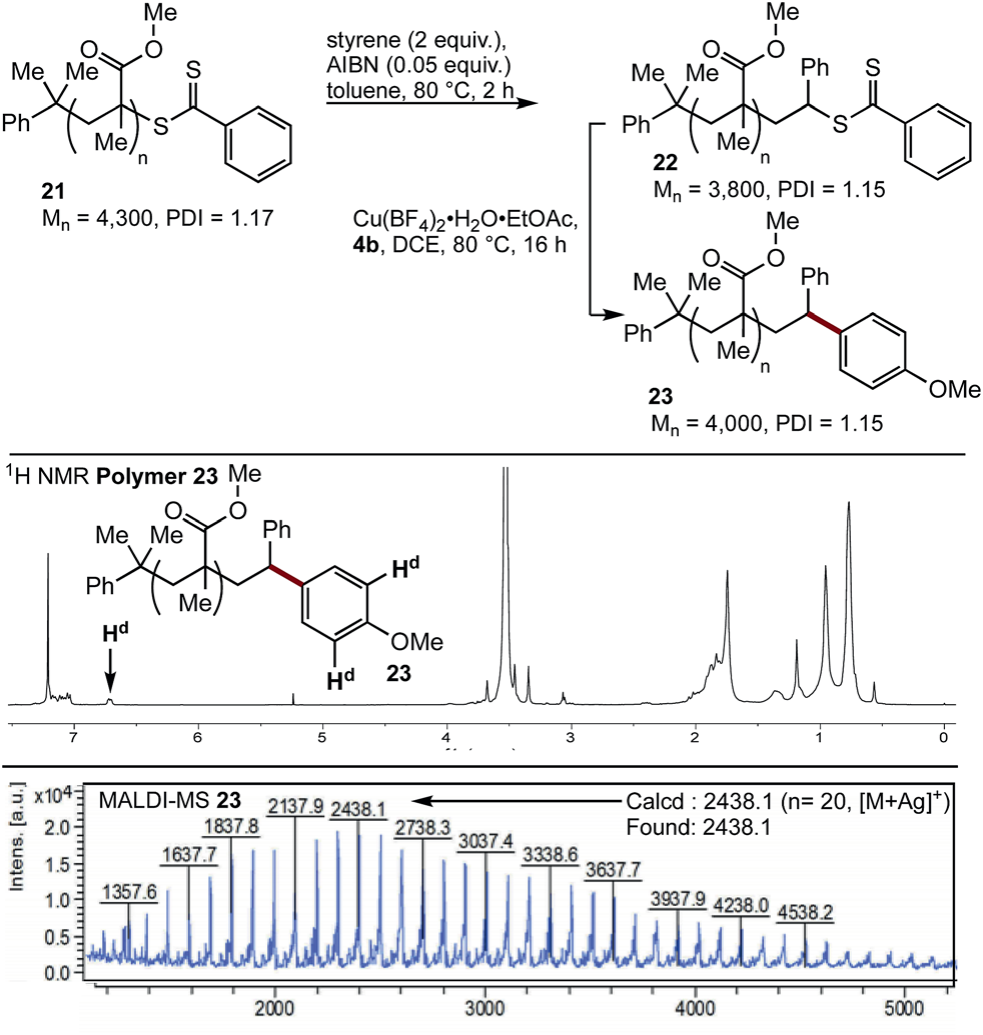
SUMI/coupling strategy for modification of polymer 22, NMR and MALDI-MS of **23**.

Having demonstrated the viability of a SUMI strategy to modify the endgroup reactivity of a methacrylate surrogate, this approach was examined with polymethylmethacrylate **21**. Single monomer insertion of styrene gave **22**, which underwent smooth cross-coupling with boronic acid **4b**, to give the endgroup modified polymethacrylate **23**. While the aryl methoxy peak was obscured in the ^1^H NMR spectra of **23**, characteristic aryl peaks could be observed at 6.7 ppm (H^d^), consistent with >95% incorporation of the end group. This was further confirmed by MALDI-MS analysis (Fig. 3).

**Fig. 4:**
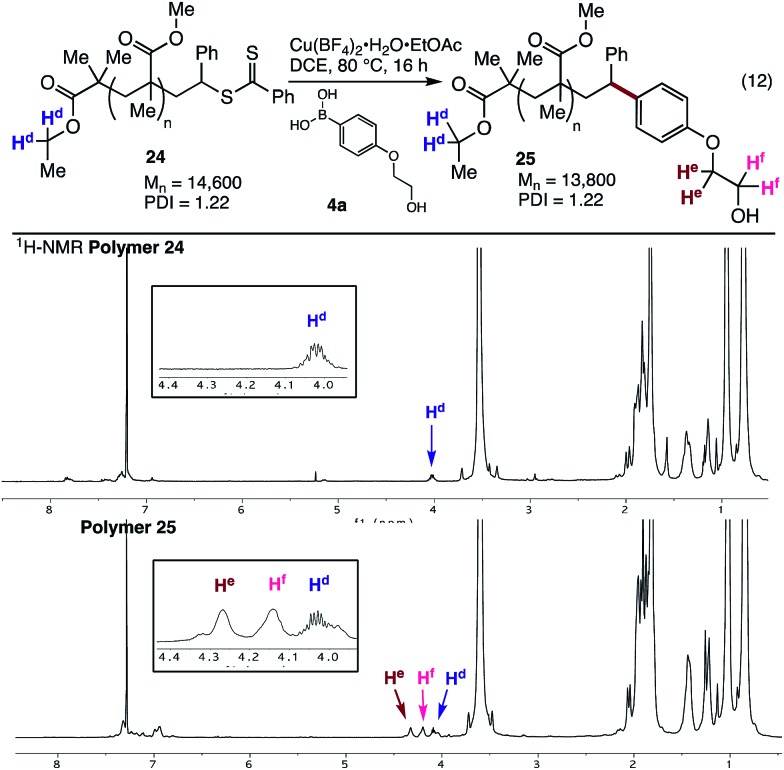
Cross-coupling with **24** and ^1^H NMR spectra of polymers **24** and **25**.

The styrene SUMI strategy was also applied to a larger polymethacrylate (*M*_n_ = 14 600), to generate polymer **24**. Coupling of this material with the ethylene glycol substituted aryl boronic acid **4a** gave the modified polymer **25** (Fig. 4). The ^1^H NMR spectra clearly shows the endgroups from both the ethyl ester (H^d^) and the ethylene glycol groups (H^e^ and H^f^, Fig. 4), while ^1^H DOESY NMR analysis shows that H^e^ and H^f^ have the same diffusion coefficient (8.4 × 10^–11^ m^2^ s^–1^) as the bulk polymer, indicating successful incorporation of the aryl boronic acid into the polymer chain.

**Fig. 5:**
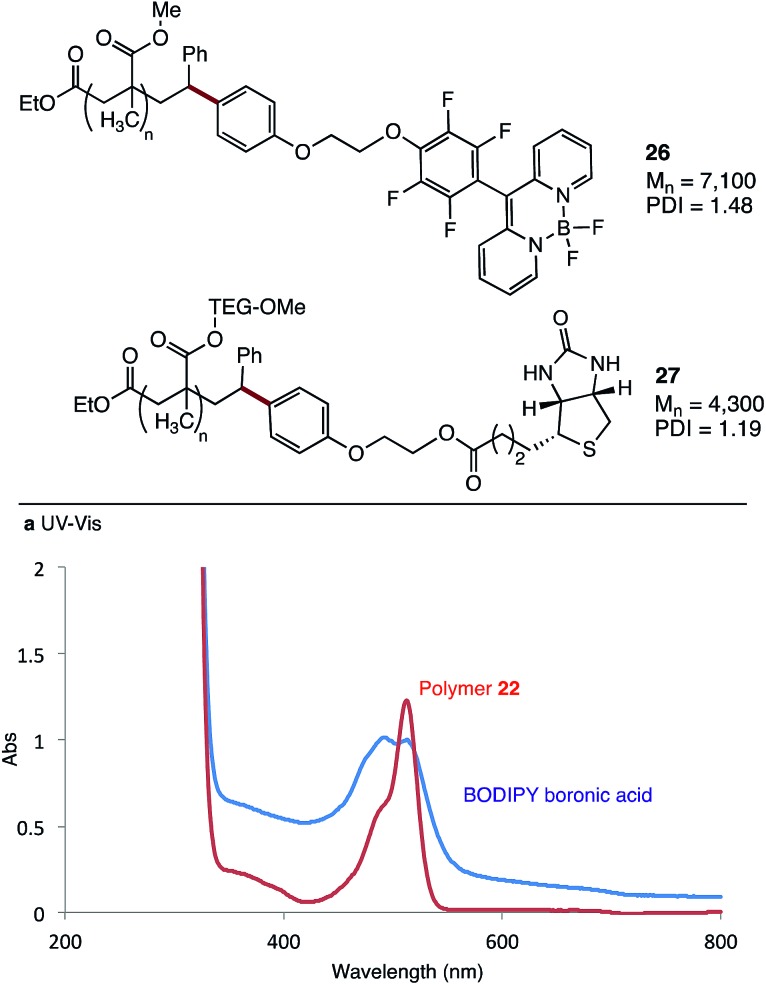
Polymer functionalisation with complex functional groups.

As a demonstration of the utility of this method, we coupled two highly functional molecules to methacrylate polymers (Fig. 5). Boronic acid **4a** was covalently linked to a BODIPY dye, before coupling with polymer **22** to give the functionalised material **26**. UV-vis analysis of the polymer shows a strong absorbance at 490–510 nm, which overlaps well with the absorbance of the BODIPY dye (Fig. 5). A similar strategy was used to synthesise polymer **27**, with a biotin functional group linked to a water soluble polymethacrylate bearing a triethylene glycol methyl ether (TEG-OMe) group. Analysis of the ^1^H NMR spectra of this polymer shows clear signals at 4.13 and 4.40 ppm, consistent with the methylene groups of the linker, along with signals at ∼6.8 ppm which we assign to the newly introduced aryl-oxy group (see ESI†).

The copper-promoted cross-coupling of RAFT polymers with aryl boronic acids is a powerful method to access functionalised polymers with controlled molecular weight and low polydispersity. This methodology has been demonstrated with a variety of polymers, including polystyrenes, and several polymethacrylates. We believe that this method will aide in the discovery of new polymers for functional materials and biological applications.

## Conflicts of interest

There are no conflicts to declare.

## Supplementary Material

Supplementary information
