# Supplementary material for: RAFT polymer cross-coupling with boronic acids
Source: Chem Sci. 2018 Jul 18;9(37):7370–5. doi: 10.1039/c8sc01862f (PMC6237125; doi:10.1039/c8sc01862f)
Supplement: Supplementary file 1 [file SC-009-C8SC01862F-s001.pdf]

Electronic Supplementary Material (ESI) for Chemical Science.
This journal is © The Royal Society of Chemistry 2018

Supporting Information
for

RAFT polymer cross coupling with boronic acids

Hartwig Golf,[a] Riley O'Shea,[a] Carl Braybrook,[b] Oliver Hutt,[b] David W. Lupton*[a] and Joel F. Hooper*[a]

a. School of Chemistry, Monash University, Clayton 3800, Victoria, Australia

b CSIRO, Research Way, Melbourne, VIC 3168, Australia.

I. General experimental **2**

II. Experimental procedures **3**

III. NMR Spectra **12**

IV. References **27**

I. General experimental

Proton (^{1}H) and carbon (^{13}C) NMR spectra were recorded in $CDCl_3$ or DMSO-d_6 on a Bruker DRX400 NMR spectrometer operating at 400 MHz for proton and 100 MHz for carbon nuclei, or a Bruker DRX600 NMR spectrometer operating at 600 MHz for proton and 150 MHz for carbon nuclei. Residual $CHCl_3$ and DMSO were used as the internal standard for ^{1}H NMR spectra (7.26 ppm for $CHCl_3$ and 2.50 ppm for DMSO). For ^{13}C NMR spectra the central peak in the $CDCl_3$ triplet (77.16 ppm), and the central peak in the DMSO-d_6 septet (39.52 ppm) were used as internal standard. NMR data recorded as follows: chemical shift (δ) [multiplicity, coupling constant(s) *J* (Hz), relative integral], where multiplicity is defined: s = singlet, d = doublet, t = triplet, q = quartet, sep = septet, m = multiplet or combinations thereof, and prefixed br = broad. Infrared spectra (υ_{max}) were recorded on a Agilent Technologies Cary 630 FTIR Spectrometer. High resolution mass spectrometry (HRMS) was performed on a Bruker BioApex 47e FTMS fitted with an Analytical electrospray source using NaI for accurate mass calibration.

Flash column chromatography was performed on silica gel (Davsil LC60A, 40-63 μm silica media) using compressed air. Concentration under reduced pressure was performed on a rotary evaporator with a water bath temperature of 40 °C.

Commercially available starting materials and reagents were purchased from Sigma-Aldrich, and AMT chemical, and were used as supplied without further purification. Diethyl ether (Et_2O) was distilled from sodium-benzophenone ketyl. Tetrahydrofuran (THF), dichloromethane (CH_2Cl_2), toluene, and acetonitrile (CH_3CN) were purified by an Innovative Technology Pure Solv MD-5 solvent purification system (SPS).

GPC analysis of polystyrene samples was performed on a EcoSEC system, eluted in 0.1 M LiBr in DMF, at an elution rate of 1 mL/min. GPC analysis of polyacrylates were performed on a PSS SDV analytical column at 40 °C, eluted in THF at 1 mL/min. Calibration was performed against polystyrene standards, and a universal calibration was applied.

Experimental procedures

1-Phenylethyl pyridine-2-carbodithioate (5a):

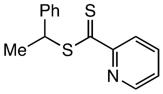

A stirred solution of 2-chloromethylpyridine (1.91 g, 15 mmol) sodium benzenesulfinate (3.70 g, 22.5mmol) tetrabutylammonium bromide (0.970 g, 3 mmol) and diazobicycloundecene (2.28g, 15 mmol) in acetonitrile (15 mL) was heated to reflux overnight. The solvent was removed *in vacuo* and the mixture was dissolved in 10 mL of dichloromethane and washed with brine (3 x 20mL). The organic layer was separated and dried ($MgSO_4$) and concentrated to give 2- ((phenylsulfonyl)methyl)pyridine, as a white solid 2.72 g.

A solution of 2-((phenylsulfonyl)methyl)pyridine (2.72g, 11.7 mmol), elemental sulfur (3.93g, 35.1 mmol) and 1-bromoethylbenzene (4.33, 23.4 mmol) in THF was stirred at room temp. overnight. To this solution potassium tert-butoxide (3.93g, 35.1 mmol) was added slowly while stirring, the solution turned from a cloudy yellow to deep purple. After 1 hour, the solvent was removed *in vacuo* and the mixture was dissolved in ethyl acetate and filtered through silica. The residue was purified by column chromatography (5% EtOAc/pet.) to give **1** as a pink oil (1-phenylethyl pyridine-2- carbodithioate, 1.2989g, yield = 43%).

^{1}H NMR (400MHz, CDCl3): δ 8.59 (dq, *J* = 4.7, 0.8Hz, 1H), 8.29 (dt, *J* = 8.0, 1 Hz, 1H), 7.77 (td, *J* = 7.5, 1.5Hz, 1H), 7.44 (m, 3H), 7.32 (m, 2H), 7.27 (m, 1H), 5.21 (q, *J*= 7 Hz, 1H), 1.81 (q, *J* = 7 Hz, 3H); ^{13}C NMR (100MHz, $CDCl_3$): δ 224.7, 156.5, 147.758, 141.5, 136.7, 128.4, 127.8, 127.4, 126.6, 122.3, 48.8, 20.8.

This data is consistent with that previously reported.[1]

1-Phenylethyl benzodithioate (5b):

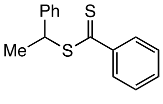

A suspension of sodium methoxide (1.08 g, 20 mmol) and sulfur (0.64 g, 20 mmol) in methanol was heated to reflux. To this solution, 2-chloromethylbenzene (1.26 g, 10 mmol) was added dropwise over an hour, and then the mixture was stirred at reflux overnight. The solvent was removed *in vacuo* and the residue was dissolved in water (20 mL) and washed with CH_2Cl_2 (20 mL). The aqueous solution was then acidified with concentrated HCl, producing a bright pink cloudy solution, and the product was extracted with CH_2Cl_2 until the pink colour was removed from the aqueous layer. The organic fractions were separated, dried ($MgSO_4$) and concentrated to give a viscous dark purple oil (benzodithioic acid). The resultant product, sodium methoxide (0.54 g, 10 mmol) and 1-bromoethylbenzene (2.22 g, 12 mmol) were dissolved in methanol and heated to reflux for 1 hour. The solvent was removed *in vacuo* and the residue purified by column chromatography (5% EtOAc/pet.) to give **2** as a red oil (1-phenylethyl benzodithioate, 0.720 g, 28%).

^{1}H NMR (400MHz, $CDCl_3$): δ 7.96 (m, 2H), 7.50 (m, 1H), 7.44 (m, 2H), 7.35 (m, 4H), 7.29 (m, 1H), 5.26 (q, J = 7 Hz, 1H), 1.82 (q, J = 7 Hz, 3H); ^{13}C NMR (100MHz, $CDCl_3$): δ 227.0, 144.9, 141.2, 132.2, 129.4, 128.6, 128.4, 128.2, 127.8, 127.6, 126.9, 50.2, 20.2.

This data is consistent with that previously reported.[2]

Preparation of $Cu(BF_4)_2.H_2O.EtOAc$

Commercially obtained $Cu(BF_4)_2.H_2O$ was dissolved in the minimum amount of a hot ethyl acetate, and the solution was filtered through a 1 cm layer of anhydrous magnesium sulfate in a fine sintered frit funnel while hot. The solvent was removed *in vacuo* to produce a pale blue amorphous solid, which was stored under an atmosphere of nitrogen.

M.p.: 86.3 °C.; FTIR: ν(cm^{-1}) 1626; 61 (sharp), 102; 18 (sharp).

1-Methoxy-4-(1-phenylethyl)benzene (6)

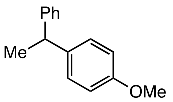

A solution of $Cu(BF_4)_2.H_2O.EtOAc$ (0.107 g, 0.45 mmol), 4- methoxyphenylboronic acid (0.045 g, 0.30 mmol) and 1-phenylethyl benzodithioate (0.025 g, 0.10 mmol) in 1,2- dichloroethane (1 mL) was heated to 80 °C for 20 hrs. The solvent was removed *in vacuo* and the product was purified by column chromatography (10% CH_2Cl_2/pet.) to give **4** as a clear oil (0.018 g, yield = 85%).

^{1}H NMR (400MHz, $CDCl_3$): δ 7.32-7.18 (m, 5H), 7.16 (d, J = 8.5 Hz, 2H), 6.85 (d, J = 9 Hz, 2H), 4.13 (q, J = 7 Hz, 1H), 3.80 (s, 3H), 1.634(d, J = 7 Hz, 3H); ^{13}C NMR (100MHz, $CDCl_3$): δ 157.8, 146.7, 138.5, 128.5, 128.294, 127.5, 125.9, 113.7, 55.12, 43.9, 22.0; GC-MS (EI^+): 212.1 (M^+, 33.8%), 197.2 (100%).

This data is consistent with that previously reported.[3]

General procedure for the synthesis of polystyrenes

A solution of ACCN (0.012 g, 0.05 mmol), and 1-phenylethyl benzodithioate (0.129 g, 0.5mmol) were dissolved in toluene (3 mL). To this mixture was added freshly distilled styrene (1.56 g, 15 mmol) and the solution was freeze degassed 3 times. The solution was heated to 90°C for 72 hours. The solvent was removed *in vacuo* and the residue was dissolved in THF (2 mL) and precipitated by pouring into 100mL of cold methanol. The polymer was filtered with a sinter funnel and the precipitation process was repeated two times, to give the polymer **7** (1.673 g, 77%).

^{1}H NMR (400MHz, CDCl3): δ 7.836 (m, 1H), 7.25-6.46 (m, 125 H), 4.88-4.68 (m, 1H), 1.84 (m, 26H), 1.43 (m, 48 H), 1.03 (m, 3H); FTIR: ν(cm^{-1}) 3026, 2920; 1601, 1493; 80, 1451, 1027, 757;

SEC (UV); Đ = 1.30, Mn = 2700.

Synthesis of polymer 8

Polystyrene **8** was prepared according to the above procedure, 1-phenylethyl benzodithioate, to give a red/pink solid.

^{1}H NMR (400MHz, $CDCl_3$): δ 7.27-6.47 (m, 156H), 3.74 (m, 3H),2.17-1.26(m, 111H), 1.05-1.70 (m, 6H); FTIR: ν(cm^{-1}) = 3025, 1625; SEC (UV); Đ = 1.23, Mn = 3000

Synthesis of polymer 9

Polystyrene **9** was prepared according to the above procedure, using 2-cyanobutan-2-yl 3,5-dimethyl-1H-pyrazole-1-carbodithioate, to give a white solid 1.67 g, 99%.

^{1}H NMR (400MHz, $CDCl_3$): δ 7.26-6.47 (m, 138H), 6.04 (m, 1H), 4.699-4.423 (m, 1H), 2.64-2.617(m, 3H), 2.23-2.21 (m, 3H), 1.88-1.44 (m, 86H), 1.00 (m, 2H), 0.88-0.86 (m, 5H); FTIR: ν(cm^{-1}) = 3025, 1625; SEC (UV); Đ = 1.24, Mn = 3200

Synthesis of polymer 10

A solution of $Cu(BF_4)_2.H_2O$.EtOAc (0.146 g, 0.62 mmol), 4- methoxyphenylboronic acid (0.038 g, 0.25 mmol) and polymer **9** (0.15 g, 0.04 mmol) in 1,2- dichloroethane (1 mL) was heated to 80 °C for 16 hrs. The solvent was removed *in vacuo* and the residue was dissolved in THF (2 mL) and precipitated by addition to cold methanol (100 mL). The polymer was isolated by filtration and the precipitation process was repeated two times, to give the polymer **7** (0.122 mg).

^{1}H NMR (400MHz, $CDCl_3$): δ 7.26-6.46 (m, 125H), 3.74 (m, 3H), 1.89 (m, 26H), 1.43 (m, 48H), 1.03 (m, 3H); FTIR: ν(cm^{-1}) = 3025, 2920, 1599, 1493, 1450, 1262, 1027, 751, 697; SEC (UV); Đ = 1.29, Mn = 3,300.

methyl 4-cyano-2-((3,5-dimethyl-1H-pyrazole-1-carbonothioyl)thio)-4-methylhexanoate (15)

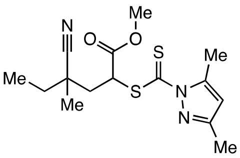

A solution of 2-cyanobutan-2-yl 3,5-dimethyl-1H-pyrazole-1-carbodithioate (1.00 g, 3.95 mmol) methyl acrylate (355µL, 7.9 mmol) and AIBN (0.5 mL, 0.2 M sol. in toluene, 0.1 mmol) in toluene (3 mL) was freeze degassed 3 times. The solution was heated to 80°C for 2 hours. The solvent was removed *in vacuo* and the residue was purified by column chromatography (5% EtOAc/pet.) to give **15**, a 1:1 mixture of diasteoisomers as a yellow oil (560 mg, 42%).

^{1}H NMR (400MHz, $CDCl_3$): δ 6.04 (s, 2H), 4.71 (ddd, J = 9.5, 7, 4 Hz, 2H), 3.71 (d, J = 6.5 Hz, 6H), 2.61 (s, 6H), 2.49 (s, J = 14, 10 Hz, 1H), 2.38 (dd, J =14.5, 9.5 Hz, 1H), 2.19 (s, 6H), 2.06 (dd, J = 14.5, 4.5 Hz, 1H), 1.87 (dd, J = 14.5, 4 Hz, 1H), 1.53-1.65 (m, 6H), 1.41 (s, 3H), 1.29 (s, 3H), 1.02 (td, J = 7.5, 4.5 Hz, 6H); ^{13}C NMR (100MHz, $CDCl_3$): δ 197.1, 170.9, 170.6, 152.5, 152.4, 136.5, 123.0, 122.9, 113.9, 113.8, 113.3, 83.6, 53.0, 48.4, 48.2, 36.8, 36.6, 33.6, 32.0, 24.9, 24.0, 23.2, 17.3, 13.7, 9.21, 9.17; MS (ESI$^+$) m/z: 245 [95], 362 [100, (M+Na)]; HRMS (ESI$^+$) Calcd. for $C_{15}H_{22}NO_3Na$: 340.1153; found: 340.1137.

ethyl 2,2-dimethyl-4-phenyl-4-((phenylcarbonothioyl)thio)butanoate (17)

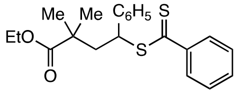
A solution of ethyl 2-methyl-2-((phenylcarbonothioyl)thio)propanoate (0.40 g, 1.5 mmol) styrene (354 µL, 3 mmol) and AIBN (354 µL, 0.2 M sol. in toluene, 0.075 mmol) in toluene (5 mL) was freeze degassed 3 times. The solution was heated to 80°C for 2 hours. The solvent was removed *in vacuo* and the residue was purified by column chromatography (10% EtOAc/pet.) to give **17** as a red oil (510 mg, 91%).

^{1}H NMR (400MHz, $CDCl_3$): δ 7.92-7.90 (m, 2H), 7.49 (tt, J = 7.5, 1, 1H), 7.42-7.51 (m, 2H), 7.35-7.30 (m, 4H), 7.26-7.23 (m, 1H), 5.29 (dd, J = 9.5, 5.5 Hz, 1H), 3.78 (dq, J = 10.5, 7 Hz, 1H), 3.68 (dq, J = 10.5, 7 Hz, 1H), 2.53 (dd, J = 14, 10 Hz, 1H), 2.40 (dd, J = 14, 5.5 Hz, 1H), 1.30 (s, 3H), 1.19 (s, 3H), 1.13 (t, J = 7 Hz, 3H); ^{13}C NMR (100MHz, $CDCl_3$): δ 176.8, 145.0, 139.8, 132.3, 128.8, 128.4, 128.3, 127.8, 126.9, 60.4, 52.7, 45.2, 42.2, 26.7, 24.7, 14.0**;** HRMS (ESI$^+$) Calcd. for $C_{21}H_{24}O_2S_2Na$: 395.1115; found: 395.1112.

ethyl 2,2-dimethyl-4-(perfluorophenyl)-4-((phenylcarbonothioyl)thio)butanoate (18)

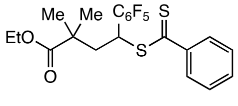
A solution of ethyl 2-methyl-2-((phenylcarbonothioyl)thio)propanoate (0.40 g, 1.5 mmol) pentafluorostyrene (415 µL, 3 mmol) and AIBN (354 µL, 0.2 M sol. in toluene, 0.075 mmol) in toluene (5 mL) was freeze degassed 3 times. The solution was heated to 80°C for 2 hours. The solvent was removed *in vacuo* and the residue was purified by column chromatography (10% EtOAc/pet.) to give **18** as a red oil (510 mg, 73%).

^{1}H NMR (400MHz, $CDCl_3$): δ 7.93-7.91 (m, 2H), 7.55-7.51 (m, 1H), 7.39-7.35 (m, 1H), 5.70 (dd, J = 9.5, 5.5, 1H), 3.95 (dq, J = 11, 7 Hz, 1H), 3.81 (dq, J = 11, 7 Hz, 1H), 2.65 (dd, J = 14.5, 9.5 Hz, 1H), 2.31 (dd, J = 14.5, 9.5 Hz, 1H), 1.33 (s, 3H), 1.21 (t, J = 14.5 Hz, 3H); ^{13}C NMR (100MHz, $CDCl_3$): δ 224.8, 176.4, 146.8 (m, CF), 144.3, 144.1, 139.5 (m, CF), 138.7 (m, CF), 136.3 (m, CF), 132.8, 128.5 (td, J = 21, 63 Hz, CF), 127.0, 114.1, 60.8, 43.28, 43.26, 41.5, 27.0, 23.9, 13.9; HRMS (ESI$^+$) Calcd. for $C_{21}H_{20}F_5O_2S_2Na$: 463.0825; found: 463.0818.

ethyl 4-(4-methoxyphenyl)-2,2-dimethyl-4-phenylbutanoate (19)

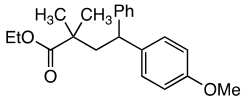 A solution of $Cu(BF_4)_2.H_2O.EtOAc$ (0.142 g, 0.60 mmol), 4- methoxyphenylboronic acid (0.059 g, 0.39 mmol) and **17** (0.050 g, 0.13 mmol) in 1,2-dichloroethane (1 mL) was heated to 80 °C for 20 hrs. The solvent was removed *in vacuo* and the product was purified by column chromatography (10% CH_2Cl_2/pet.) to give **19** as a clear oil (0.019 g, yield = 90%).

^1H NMR (400MHz, $CDCl_3$): δ 7.21 (d, J = 8.5, 1H), 7.65 (d, J = 8 Hz, 1H), 7.52-7.48 (m, 1H), 7.45-7.40 (m, 1H), 7.29 (d, J = 7 Hz, 1H), 7.03 (d, J = 1H), 6.96-6.92 (m, 2H), 6.80-6.76 (m, 2H), 4.53 (d, J = 3 Hz, 1H), 4.18-4.02 (m, 2H), 3.79 – 3.76 (m, 3H), 1.25 (s, 3H), 1.14 (s, 3H), 1.15 (t, J = 7 Hz, 2H); ^{13}C NMR (100MHz, $CDCl_3$): δ 177.2, 158.1, 148.2, 144.3, 138.6, 128.6, 128.2, 127.9, 123.6, 123.0, 120.8, 119.8, 113.9, 61.2, 60.6, 55.3, 52.4, 46.8, 22.5, 22.1, 14.0; HRMS (ESI^+) Calcd. for $C_{21}H_{30}ON$ (M + NH_4): 344.2226; found: 344.2189.

Synthesis of polymer 21

A solution of AIBN (0.92 mL, 0.2 M in toluene, 0.018 mmol), and 1-phenylethyl benzodithioate (1.0 g, 3.67 mmol) were dissolved in toluene (5 mL). To this mixture was added freshly distilled methyl methacrylate (11.7 mL, 110 mmol) and the solution was freeze degassed 3 times. The solution was heated to 80°C for 16 hours. The product was precipitated by pouring into 500 mL of ether. The polymer was filtered with a sinter funnel and the precipitation process was repeated two times with THF/ether, to give the polymer 1**7** (7.36 g) as a red solid.

^1H NMR (400MHz, $CDCl_3$): δ 7.84-7.80 (m, 2H), 7.47-7.43 (m, 1H), 7.31-7.27 (m, 2H), 7.21-7.17 (m, 4H), 7.01-7.05 (m, 1H), 3.71-3.34 (m, 114H), 1.96-1.69 (m, 70H) 1.40-1.12 (m, 30H), 1.01-0.54 (m, 118H); SEC (UV); Đ = 1.17, Mn = 4,300.

Synthesis of polymer 22

A solution of polymer **21** (1.0 g, 0.29 mmol) styrene (65 µL, 58 mmol) and AIBN (72 µL, 0.2 M sol. in toluene, 0.014 mmol) in toluene (3 mL) was freeze degassed 3 times. The solution was heated to 80°C for 3 hours. The solvent was removed *in vacuo* and the residue was purified by gel permeation chromatography on Sephadex LH-20 resin, eluted in 1:1 MeOH/CH_2Cl_2 to give polymer **22** (0.957 g) as a pale pink solid.

^1H NMR (400MHz, $CDCl_3$): δ 7.84-7.79 (m, 2H), 7.45-7.37 (m, 2H), 7.31-7.19 (m, 9H), 7.09-7.06 (m, 2H), 5.15 (m, 1H), 3.51-3.35 (m, 98H), 1.91-1.71 (m, 61H), 1.51-1.34 (m, 14H), 1.19-1.14 (m, 37H), 0.81-0.69 (m, 62H); SEC (UV); Đ = 1.15, Mn = 4,800

Synthesis of polymer 23

A solution of $Cu(BF_4)_2.H_2O.EtOAc$ (0.111 g, 0.46 mmol), 4- methoxyphenylboronic acid (0.031 g, 0.31 mmol) and **18**

(0.250 g, 0.05 mmol) in 1,2-dichloroethane (2 mL) was heated to 80 °C for 16 hrs. The solvent was removed *in vacuo* and the product was purified by gel permeation chromatography on Sephadex LH-20 resin, eluted in 1:1 MeOH/CH_2Cl_2 to give polymer **23 (**0.237 g) as a colourless solid.

^{1}H NMR (400MHz, $CDCl_3$): δ7.25-7.09 (m, 12H), 6.77-6.75 (m, 2H), 3.73 (m, 2H), 3.51-3.40 (m, 196H), 2.07-1.80 (m, 130H), 1.42-0.77 (m, 240H); SEC (UV); Đ = 1.15, Mn = 5,000

Synthesis of polymer 24

A solution of polymethyl methacrylate (3.0 g, 0.75 mmol) styrene (170 µL, 1.5 mmol) and AIBN (188 µL, 0.2 M sol. in toluene, 0.038 mmol) in toluene (5 mL) was freeze degassed 3 times. The solution was heated to 80°C for 3 hours. The product was precipitated by pouring into 100 mL of ether. The polymer was filtered with a sinter funnel and the precipitation process was repeated two times with THF/ether, to give the polymer **24** as a pale red solid (3.01 g).

^{1}H NMR (400MHz, $CDCl_3$): 7.84-7.79 (m, 3H), 7.46-7.37 (m, 2H), 7.31-7.23 (m, 5H), 5.14 (m, 1H), 4.06-3.98 (m, 4H), 3.74-3.32 (m, 481H), 2.10-1.54 (m, 294H), 1.43-0.49 (m, 542H); SEC (UV); Đ = 1.22, Mn = 14,600.

Synthesis of polymer 25

A solution of $Cu(BF_4)_2.H_2O.EtOAc$ (0.037 g, 0.15 mmol), **4a** (0.018 g, 0.10 mmol) polymer **24** (0.250 g, 0.017 mmol) in 1,2-dichloroethane (2 mL) was heated to 80 °C for 16 hrs. The solvent was removed *in vacuo* and the product was purified by gel permeation chromatography on Sephadex LH-20 resin, eluted in 1:1 MeOH/CH_2Cl_2 to give **25** (0.194 g) as a colourless solid.

^{1}H NMR (400MHz, $CDCl_3$): δ 7.38-7.28 (m, 6H), 6.99-6.92 (m, 3H), 4.33-4.30 (m, 2H), 4.22-4.18 (m, 2H), 4.12-4.03 (m, 2H), 3.78-3.15 (m, 491H), 2.07-1.74 (m, 300H), 1.44-0.92 (m, 570H); SEC (UV); Đ = 1.22, Mn = 13,800.

5-(pentafluorophenyl)-dipyrromethane (S1)

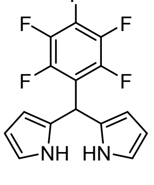

A mixture of pyrrole (150 mL, 2.16 mol) and pentafluoro-benzaldehyde (7.30 mL, 59.0 mmol) were stirred for 15 min. TFA (0.45 mL, 5.84 mmol) was added and the mixture stirred for 20 min. at room temperature. The excess pyrrole was evaporated under reduced pressure at 60 °C and the resulting oil purified by column chromatography (50% DCM/pet.) and recrystallyzation (DCM/*n*-penatane) to obtain the dipyrromethane **S1** as a colorless solid (12.8 g, 70%).

^{1}H-NMR (400 MHz, $CDCl_3$): δ 8.09 (br s, 2H), 6.71-6.73 (m, 2H), 6.15-6.19 (m, 2H), 6.01-6.05 (m, 2H), 5.89 (s, 1H, CH), ppm. ^{19}F-NMR (376 MHz, $CDCl_3$): δ -141.39 (d, J = 22.4 Hz, 2F), -155.67 (t, J = 20.7 Hz), -161.05, -161.22 (m, 2F)

This data is consistent with that previously reported.[4]

pinacolato-borane dipyrromethane (S2)

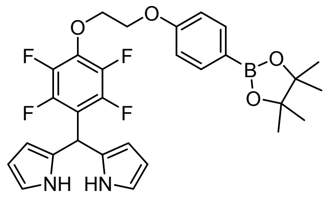

To a solution of 5-(pentafluorophenyl)-dipyrromethane **S1** (2.70 g, 8.70 mmol) in THF (5.0 mL) at 0 °C was added NaH (60% in mineral oil, 0.70 g, 17.4 mmol). After stirring for 10 min. at 0 °C this solution was added dropwise into a mixture of 2-(4-(4,4,5,5-tetramethyl-1,3,2-dioxaborolan-2-yl)phenoxy)ethan-1-ol (4.60 g, 17.4 mmol) and NaH (60% in mineral oil, 0.70 g, 17.4 mmol) in THF (10 mL) at 0 °C. The reaction mixture was allowed to reach room temperature upon complete addition and was stirred for 16 h. The reaction was quenched by dropwise addition of water at 0 °C followed by extraction with DCM. The organic phase was washed 3x with water, brine solution and dried over sodium sulfate. The solvent was removed *in vacuo* and the residue was purified by column chromatography (DCM) to give dipyrromethane **S2** as a yellow solid (1.75 g, 36%).

^{1}H-NMR (400 MHz, $CDCl_3$): δ 8.14 (br s, 2H), 7.76 (d, J = 8.6 Hz, 2H), 6.88 (d, J = 8.6 Hz, 2H), 6.71 (s, 2H), 6.18 (q, J = 2.9 Hz, 2H), 6.05 (s, 2H), 5.88 (s, 1H), 4.60-4.50 (m, 2H), 4.38-4.27 (m, 2H), 1.36 (s, 12H); ^{13}C-NMR (101 MHz, $CDCl_3$): 160.9, 146.3, 143.8, 142.9 – 142.4 (m), 140.2 (d, J = 16.0 Hz), 136.6, 128.7, 118.0, 113.9, 108.6, 107.5, 83.65, 73.0, 66.8, 33.1, 24.9; ^{11}B-NMR (128 MHz, $CDCl_3$): δ 30.2 (br s); ^{19}F-NMR (376 MHz, $CDCl_3$): -143.1 (d, J = 16.0 Hz), -156.05 (dd, J = 22.1, 8.6 Hz); HRMS (ESI$^+$) Calcd. for $C_{29}H_{29}BF_4N_2O_4$: 556.2157; found: 556.2166.

pinacolato-borane BODIPY (S3)

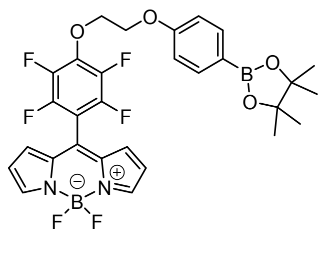

Ether-linked dipyrromethane **S2** (1.0 g, 1.79 mmol) was dissolved in DCM (100 mL) under nitrogen atmosphere and

DDQ (0.34 g, 1.52 mmol) was added. After 5 min. DIPEA (1.86 mL, 10.6 mmol) was added via canula, stirring continued for 15 min. followed by addition of $BF_3O(Et)_2$ (2.07 mL, 16.8 mmol) and stirring for 20 min. at room temperature. The reaction was quenched with water. Extraction was carried out with DCM, the organic phase washed 3x with water, brine solution and dried over sodium sulfate. The solvent was removed *in vacuo* and the dark colored residue purified by column chromatography (DCM) to give functionalized BODIPY **S3** as a red-green solid (460 mg, 50%).

^{1}H NMR (400 MHz, $CDCl_3$) δ 7.87 (br s, 2H), 7.68 (br s, 2H), 6.80 (br s, 2H), 6.73 (br s, 2H), 6.47 (br s, 2H), 4.64 (br s, 2H), 4.31 (br s, 2H), 1.25 (s, 12H, CH_3); ^{13}C NMR (101 MHz, $CDCl_3$) δ 160.7, 146.2, 136.7, 135.1, 130.7, 119.5, 113.8, 83.7, 73.1, 66.8, 24.9 (fluorinated carbons not detected); ^{11}B NMR (128 MHz, $CDCl_3$) δ 31.0 (br s, BOH), 0.21 (t, J = 28.2 Hz, BF_2); ^{19}F-NMR (376 MHz, $CDCl_3$): -138.55 – -138.79 (m, 2F, Ar-F), -144.99 (m, 2F, BF_2), -154.51 – -155.19 (m, 2F, Ar-F); HRMS (ESI$^+$) Calcd. for $C_{29}H_{26}B_2F_6N_2O_4$: 602.1983; found: 602.1932.

BODIPY boronic acid (S4)

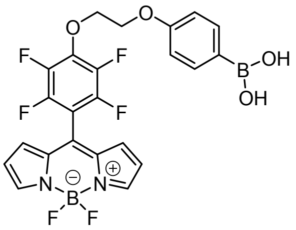

Pinacolato-borane BODIPY **S3** (65.1 mg, 0.11 mmol) was dissolved in a mixture of acetone/water (10 mL, 1:1 v/v) followed by addition of $NaIO_4$ (69.3 mg, 0.32 mmol), NH_4Ac (24.9 mg, 0.32 mmol) and stirring for 5 h at 50 °C. The mixture was allowed to cool to room temperature and acetone was removed *in vacuo*. Extraction was carried out with DCM, the organic phase washed 3x with brine solution and dried over sodium sulfate. The solvent was removed *in vacuo* and the dark purple colored crude purified by column chromatography (70% EtOAc/pet.) to give functionalized BODIPY boronic acid **S4** as a purple solid (32 mg, 57%).

^{1}H NMR (400 MHz, $CDCl_3$) δ 8.19 (d, J = 8.6 Hz, 2H), 7.96 (s, 2H), 7.02 (d, J = 8.6 Hz, 2H), 6.84 (d, J = 4.1 Hz, 2H), 6.56 (d, J = 3.9 Hz, 2H), 4.81-4.75 (m, 2H, CH_2), 4.50-4.43 (m, 2H, CH_2); ^{13}C NMR (101 MHz, $CDCl_3$) δ 162.0, 146.4, 137.8, 135.7, 130.8, 119.7, 114.2, 73.2, 67.1 (fluorinated carbons not detected); ^{19}F NMR (377 MHz, $CDCl_3$) δ -138.4 – -138.6 (m, 2F, Ar-F), -145 (dd, J = 56.6, 28.3 Hz, 2F, BF_2), -154.20 – -155.41 (m, 2F, Ar-F); HRMS (ESI$^+$) Calcd. for $C_{23}H_{16}B_2F_6N_2O_4$: 520.1200; found: 520.1237.

pinacolato-borane biotin ester (S5)

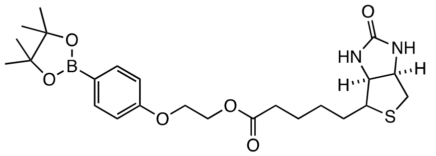

A flame dried and nitrogen purged 250 mL reaction flask was loaded with biotin (1.0 g, 4.09 mmol), 4-DMAP (50 mg, 0.40 mmol) and EDCI (1.27 g, 8.18 mmol). The reaction vessel was several times evacuated and purged with nitrogen. Dry DMF (30 mL) was added via canula and the reaction mixture cooled down to 0 °C. 2-(4-(pinacolatoboron)phenoxy)ethanol was loaded in a secon reaction tube, vacuum dried, nitrogen purged, dissolved in dry DMF (5.0 mL) and added over 2 h dropwise via canula into the biotion mixture at 0 °C. Upon complete addition, the reaction mixture was allowed to warm to room temperature and stirred for 48 h under nitrogen atmosphere. The solvent was removed *in vacuo,* the residue dissolved in DCM, washed with NaOH sol. (1M) and four times with water. DCM was removed under reduced pressure, the crude dissolved in THF and precipitated in excess petrol ether. Purification was carried out by column chromatography (10% MeOH/DCM) to give biotin ester S5 as a white solid (1.51 g, 73%).

^{1}H NMR (400 MHz, DMSO-d_6) δ 7.45 (d, J = 8.9 Hz, 2H), 6.94 (d, J = 9.0 Hz, 2H), 6.38 (d, J = 25.1 Hz, 2H), 4.37 – 4.31 (m, 2H), 4.22 – 4.14 (m, 2H), 3.05 (ddd, J = 8.3, 6.1, 4.4 Hz, 1H), 2.81 (dd, J = 12.4, 5.1 Hz, 1H), 2.58 (d, J = 12.4 Hz, 1H), 2.51 (p, J = 1.9 Hz, 4H), 2.33 (t, J = 7.4 Hz, 2H), 2.09 (s, 12H), 1.67 – 1.40 (m, 4H), 1.39 – 1.29 (m, 2H). ^{13}C NMR (101 MHz, DMSO-d_6) δ 13C NMR (101 MHz, CDCl3) δ 173.6, 158.4, 136.6, 132.3, 129.5, 121.2, 116.5, 114.6, 105.0, 100.1, 70.6, 65.9, 61.9, 60.1, 55.5, 40.5, 33.7, 28.3, 24.9, 24.8; HRMS (ESI$^+$) Calcd. for $C_{26}H_{39}BN_2O_6S$: 518.2622; found: 518.2611.

biotin boronic acid (S6)

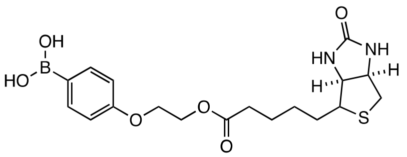

To a solution of the pinacol ester **S5** (0.50 g, 1.0 mmol) in CH_2Cl_2 (20 mL) was added KHF_2 (0.27 g, 3.5 mmol). After stirring for 8 hrs, the precipitate was collected by filtration to give the potassium fluoroborate salt. This solid was dissolved in 5% aqueous MeCN/ 0.1 M formic acid (100 mL) and stirred at room temperature for 2 hrs. Water was added (200 mL) and the aqueous phase was extracted with 3:1 $CHCl_3$/^{i}PrOH (5 x 50 mL). The combined organic extracts were dried ($MgSO_4$) and concentrated to yield the boronic acid as a colourless solid (0.15 g, 36%).

^{1}H NMR (400 MHz, DMSO-d_6) δ 7.75-7.73 (m, 2H), 6.92-6.90 (m, 2H), 4.36 (dd, J = 5.5, 3.5 Hz, 2H), 4.29 (dd, J = 7.5, 4.5 Hz, 1H), 4.20 (t, J = 4.5 Hz, 2H), 4.10 (dd, J = 7.5, 4.5 Hz, 1H), 3.04 (ddd, J = 8.5, 6, 6 Hz, 1H), 2.80 (dd, J = 12.5, 5 Hz, 1H), 2.34 (t, J = 7.5 Hz, 2H), 1.64-1.31 (m, 6H), 1.05 (d, J = 6 Hz, 2H); ^{13}C NMR (101 MHz, DMSO-d_6) δ

173.3, 163.2, 160.4, 158.6, 136.4, 130.0, 121.3, 115.0, 114.0, 66.0, 62.8, 61.5, 59.7. 55.8, 40.3, 33.7, 31.2, 28.5, 28.4, 25; HRMS (ESI$^+$) Calcd. for $C_{16}H_{26}BN_2O_6S$: 509.1605; found: 509.1607.

Synthesis of polymer 26

A solution of $Cu(BF_4)_2.H_2O.EtOAc$ (0.060 g, 0.25 mmol), **S4** (0.087 g, 0.16 mmol) polymer **22** (0.10 g, 0.028 mmol) in 1,2-dichloroethane (2 mL) was heated to 80 °C for 16 hrs. The solvent was removed *in vacuo* and the product was purified by gel permeation chromatography on Sephadex LH-20 resin, eluted in 1:1 MeOH/CH_2Cl_2 to give **26** (0.85 g) as a black solid.

^{1}H NMR (400MHz, $CDCl_3$): δ 7.92-7.18 (m, 12H), &.09-&.07 (m, 2H), 4.33-4.20 (m, 2H), 3.97-3.76 (m, 2H), 3.62-3.34 (m, 121H), 2.05-1.34 (m, 106H), 1.18-0.77 (m, 148H); SEC (UV); Đ = 1.48, Mn = 7,100

Synthesis of polymer 23

A solution of $Cu(BF_4)_2.H_2O.EtOAc$ (0.060 g, 0.25 mmol), **S4** (0.065 g, 0.16 mmol) TEG methacrylate polymer (0.10 g, 0.028 mmol) in 1,2-dichloroethane (2 mL) was heated to 80 °C for 16 hrs. The solvent was removed *in vacuo* and the product was purified by gel permeation chromatography on Sephadex LH-20 resin, eluted in 1:1 MeOH/CH_2Cl_2 to give **23** (0.101 g) as a colourless solid.

^{1}H NMR (400MHz, $CDCl_3$): δ 7.32-7.17 (m, 10H), 7.07-7.05 (m, 2H), 6.9106.85 (m, 2H), 4.38-4.35 (m, 2H), 4.12-43.95 (m, 69H), 3.69-3.43 (m, 345H), 3.37-3.28 (m 101H), 2.06-1.69 (m, 73H), 1.32-0.74 (m, 129H); SEC (UV); Đ = 1.19, Mn = 4,300

NMR Spectra

Polymer 7
^{1}H NMR ($CDCl_3$, 400 MHz)

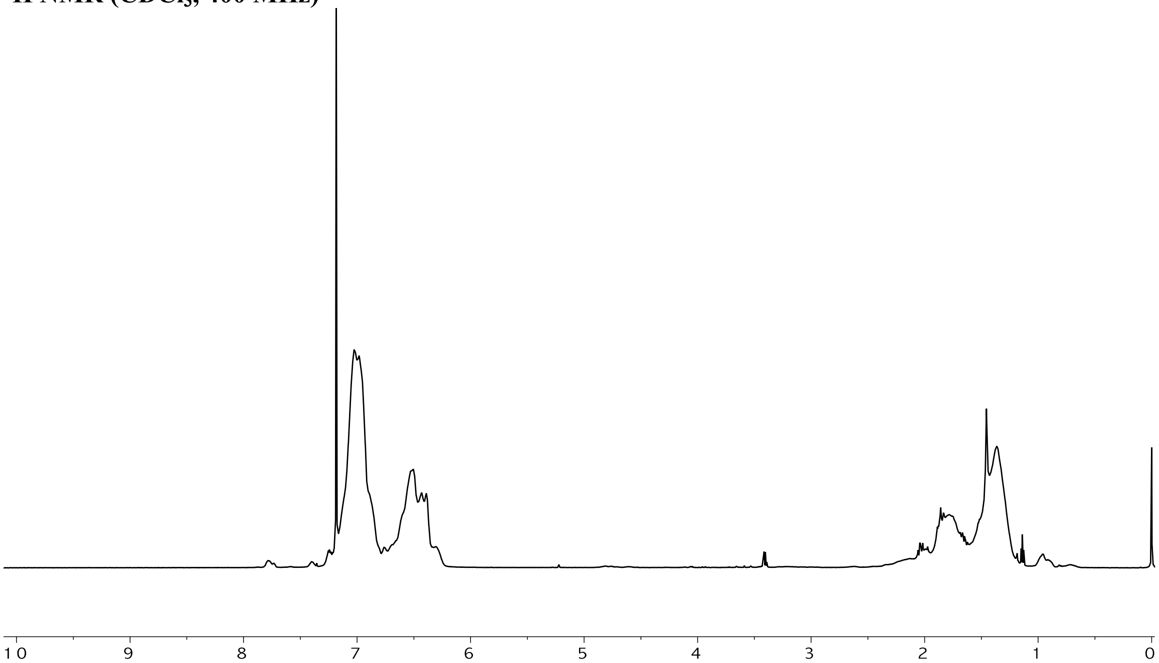

Polymer 8
^{1}H NMR ($CDCl_3$, 400 MHz)

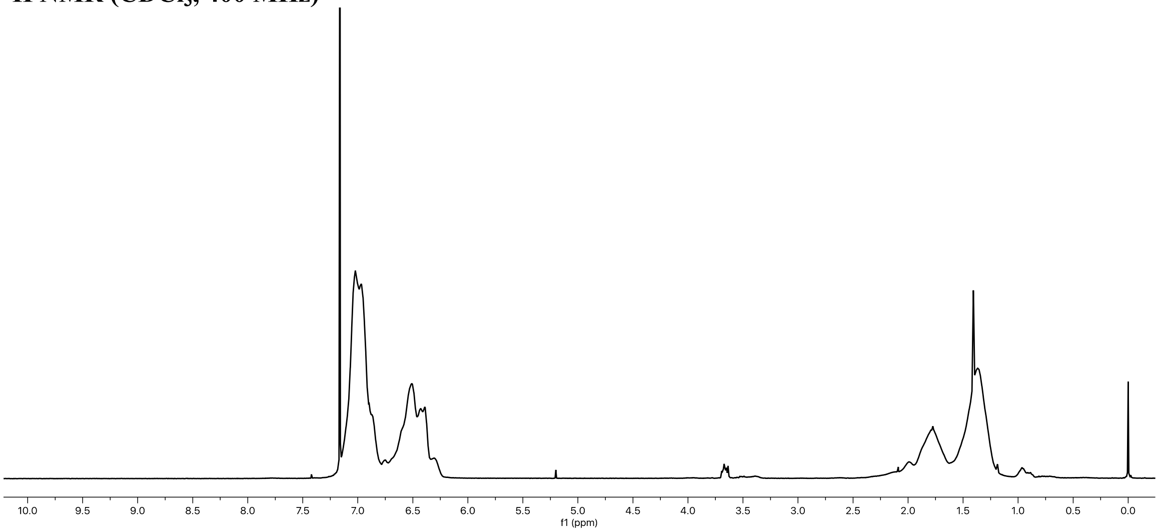

Polymer 9
^{1}H NMR ($CDCl_3$, 400 MHz)

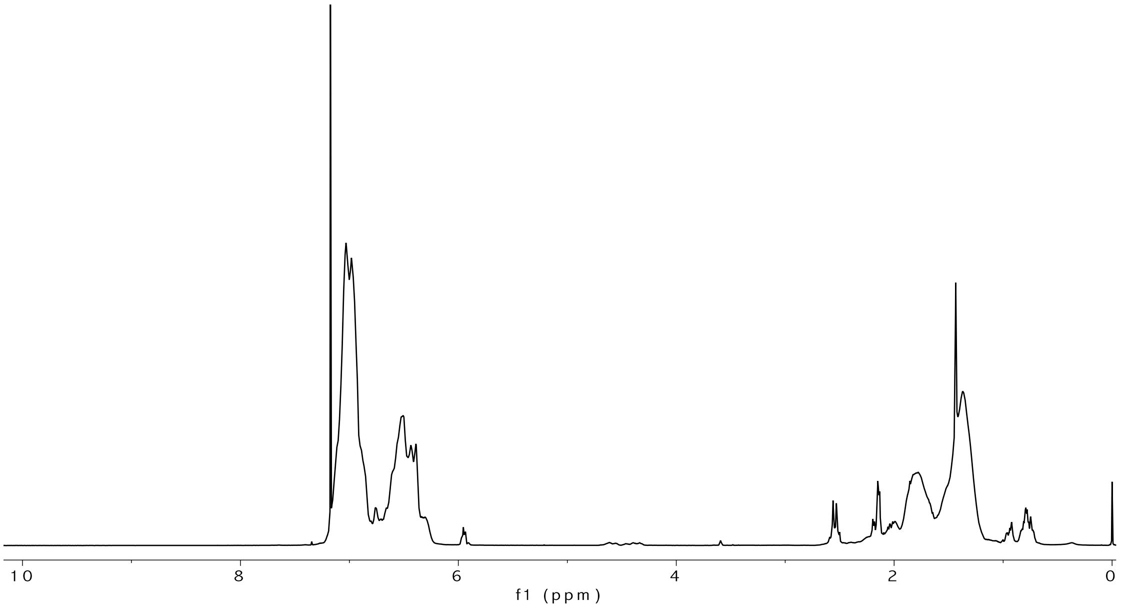

Polymer 10
^{1}H NMR ($CDCl_3$, 400 MHz)

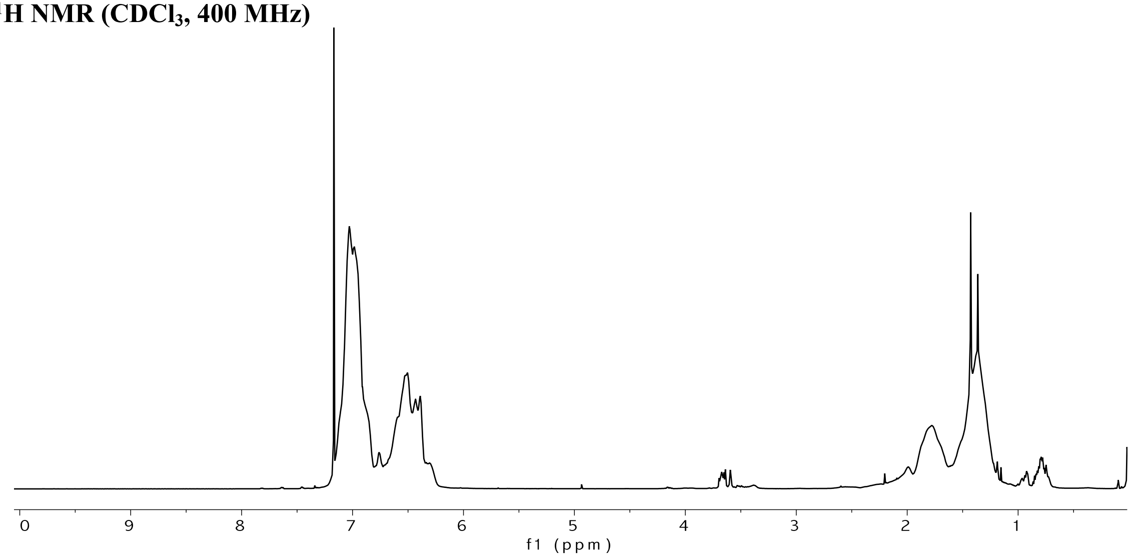

Polymer 11

^{1}H NMR ($CDCl_3$, 400 MHz)

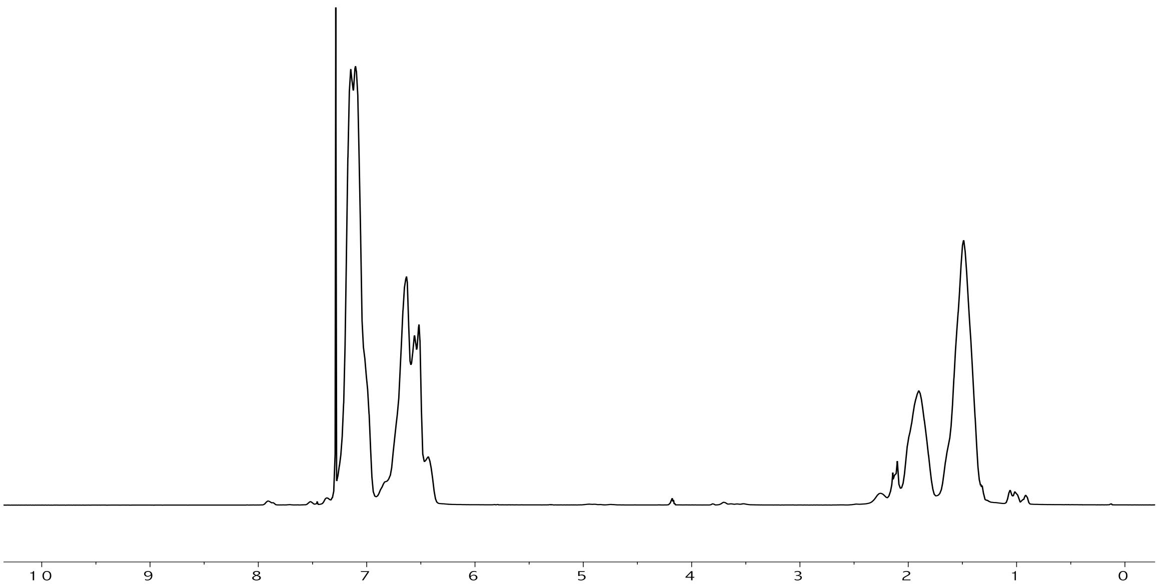

Polymer 12

^{1}H NMR ($CDCl_3$, 400 MHz)

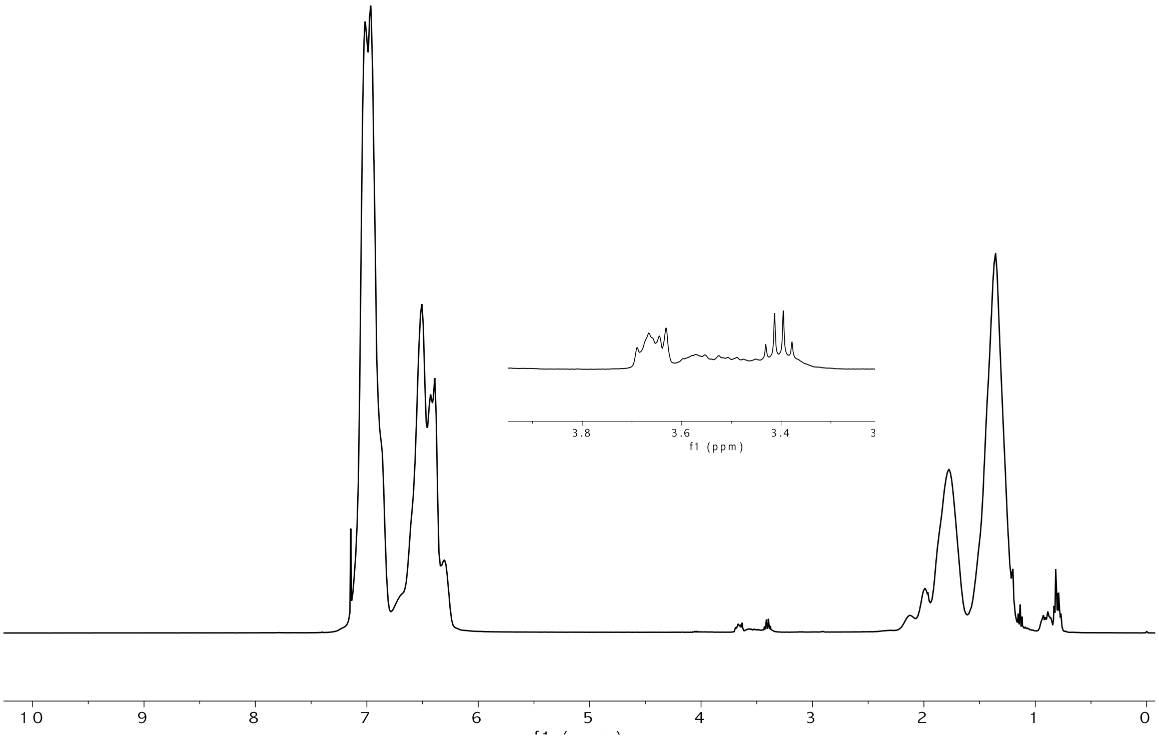

Polymer 13
^{1}H NMR (CDCl$_3$, 400 MHz)

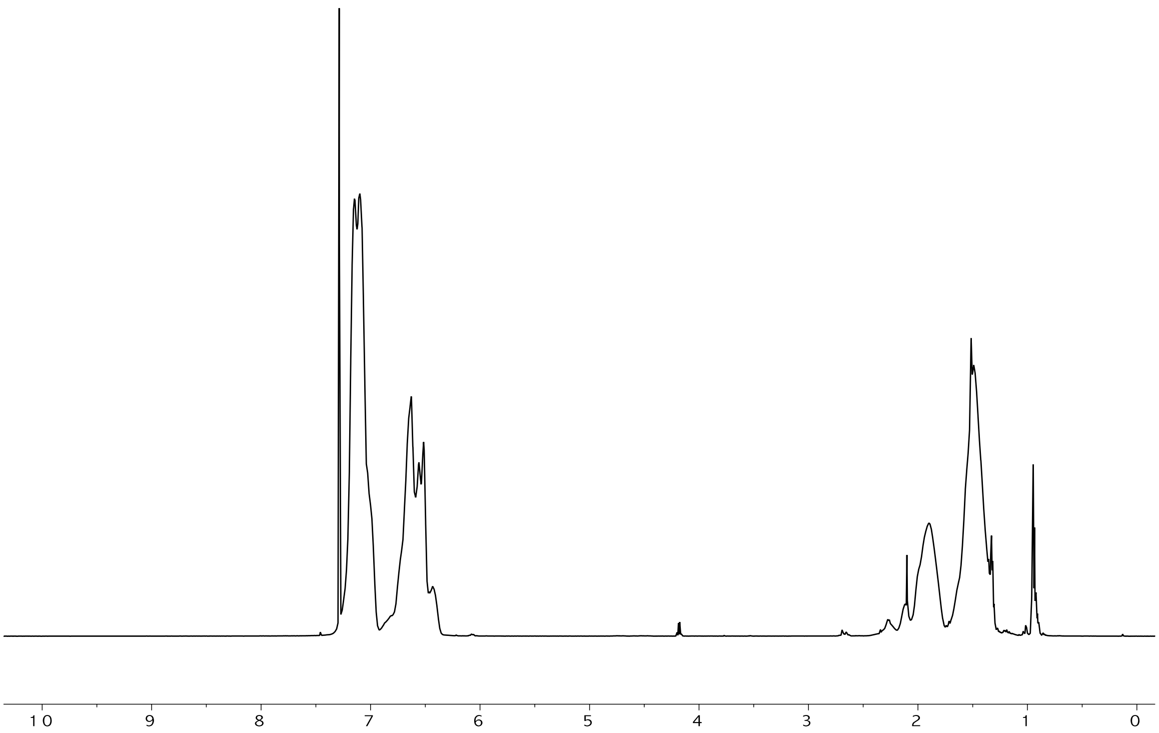

Polymer 14

^{1}H NMR (CDCl$_3$, 400 MHz)

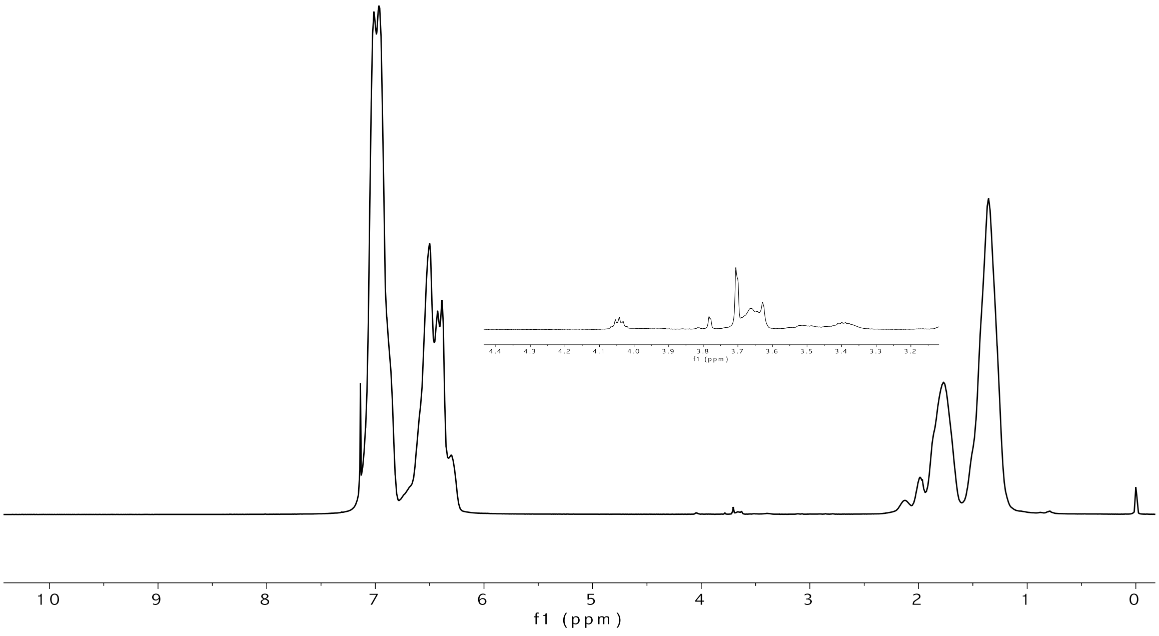

15

^{1}H NMR ($CDCl_3$, 400 MHz)

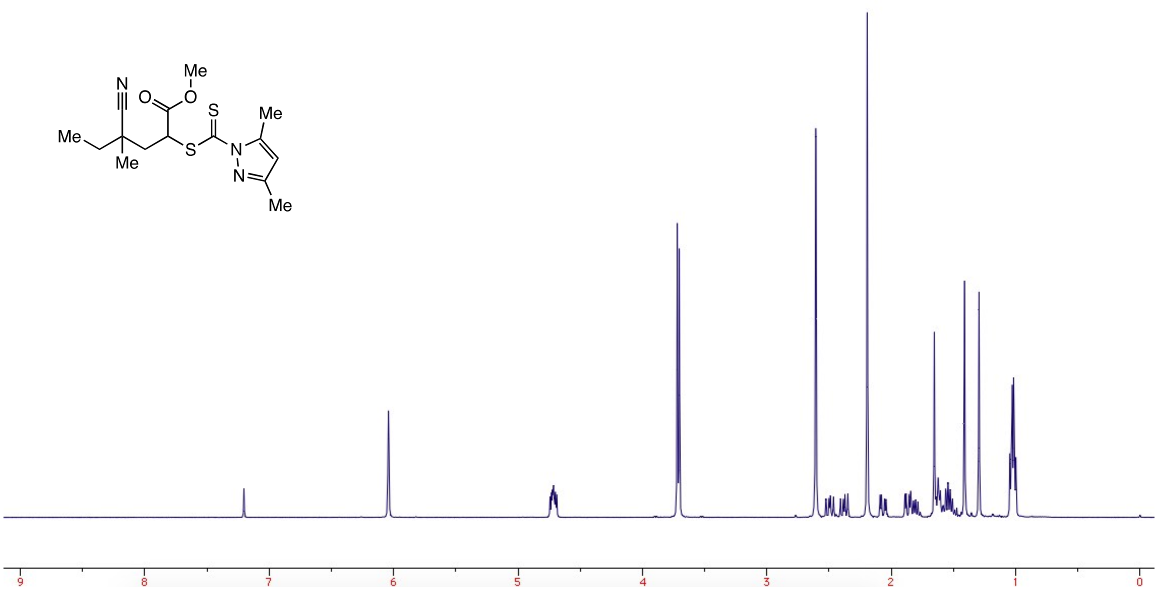

^{13}C NMR ($CDCl_3$, 100 MHz)

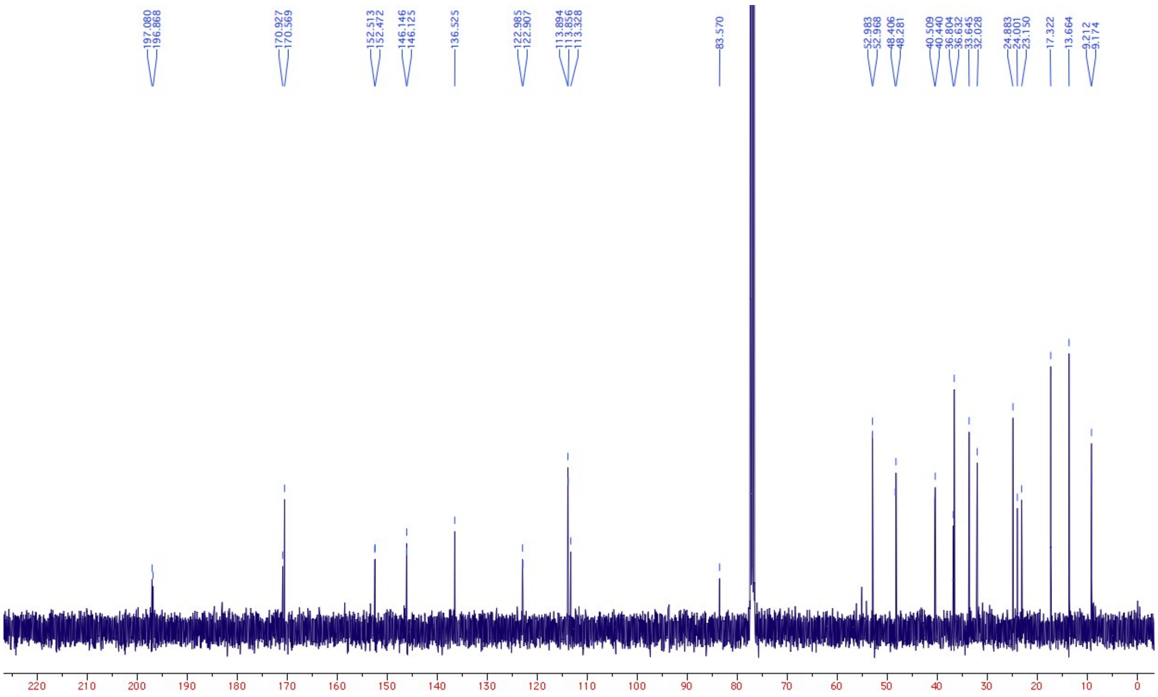

17

^{1}H NMR ($CDCl_3$, 400 MHz)

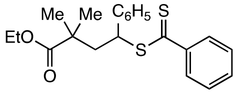

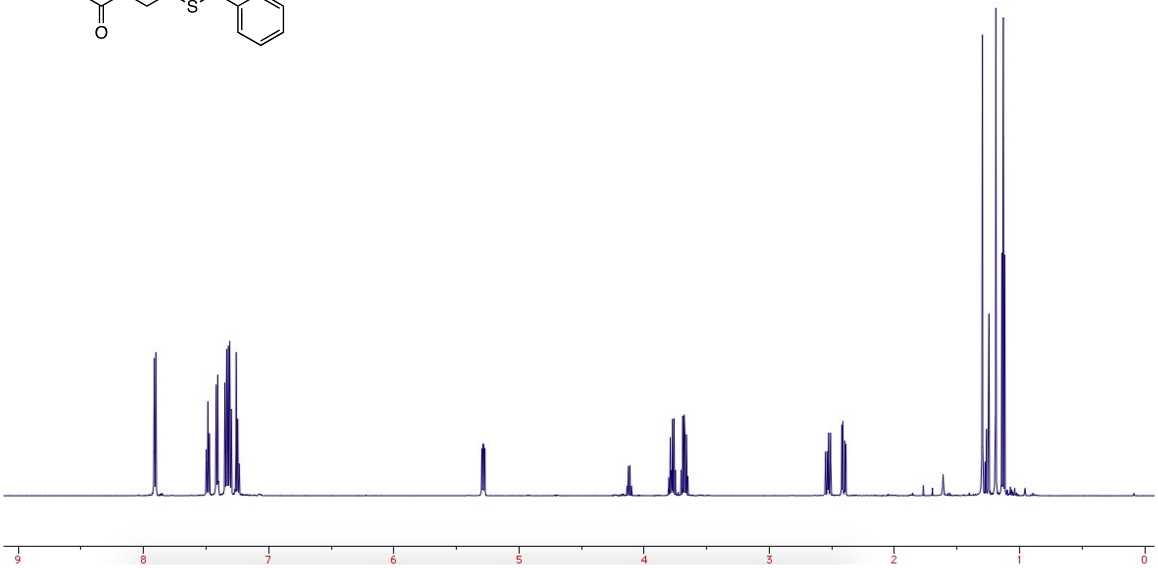

^{13}C NMR ($CDCl_3$, 100 MHz)

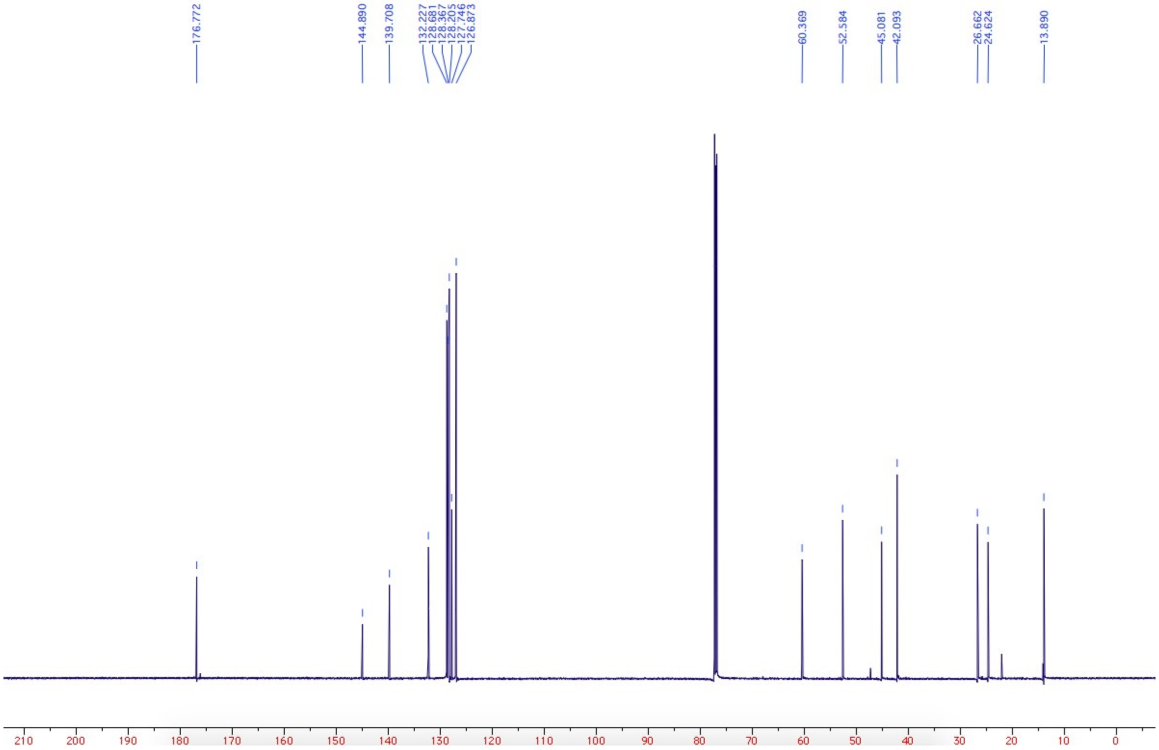

18

^{1}H NMR ($CDCl_3$, 400 MHz)

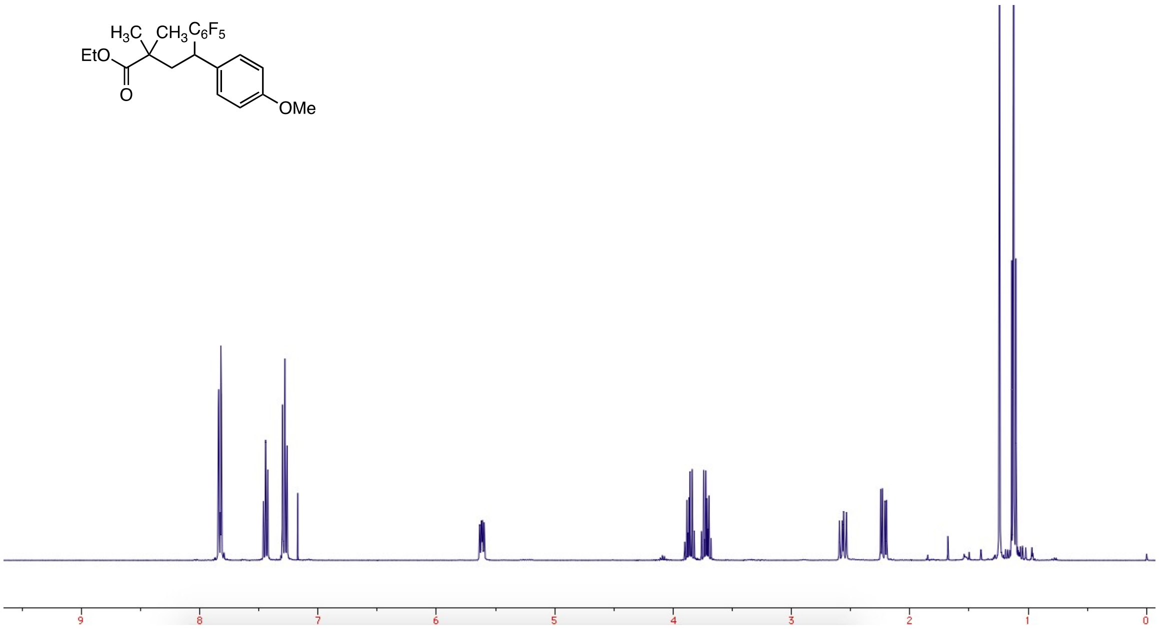

^{13}C NMR ($CDCl_3$, 100 MHz)

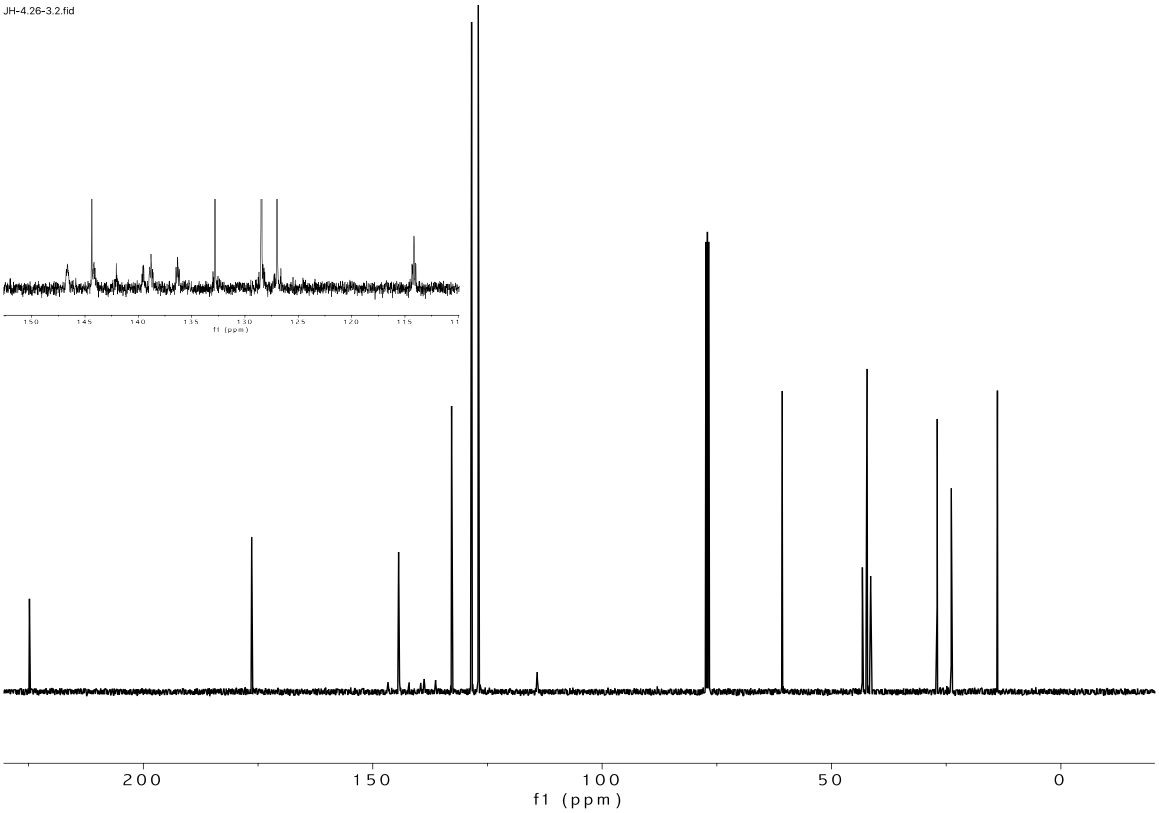

19

^{1}H NMR (CDCl$_3$, 400 MHz)

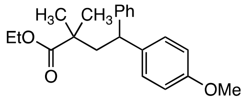

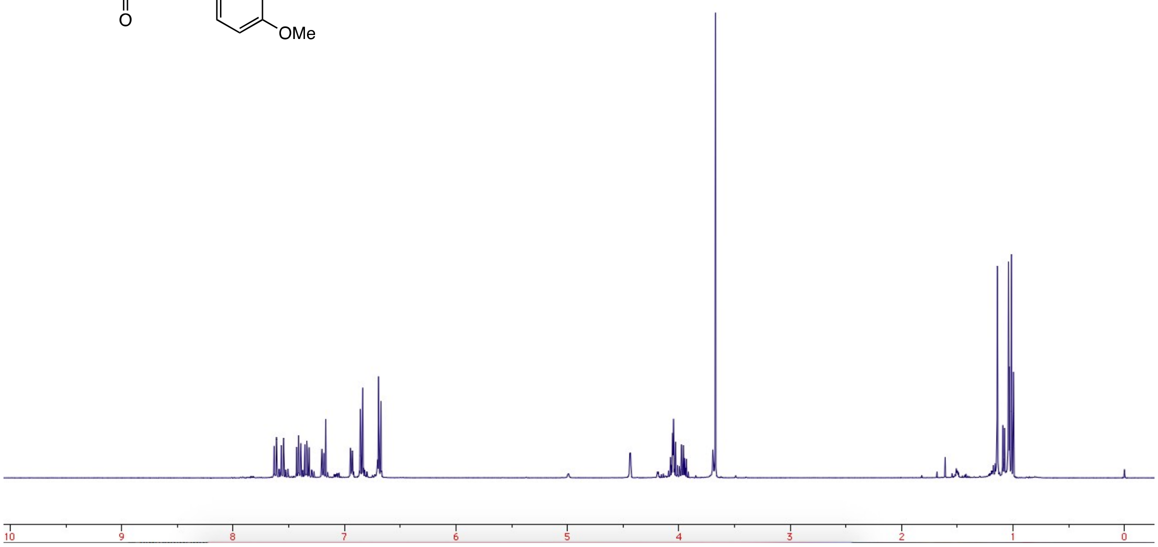

^{13}C NMR (CDCl$_3$, 100 MHz)

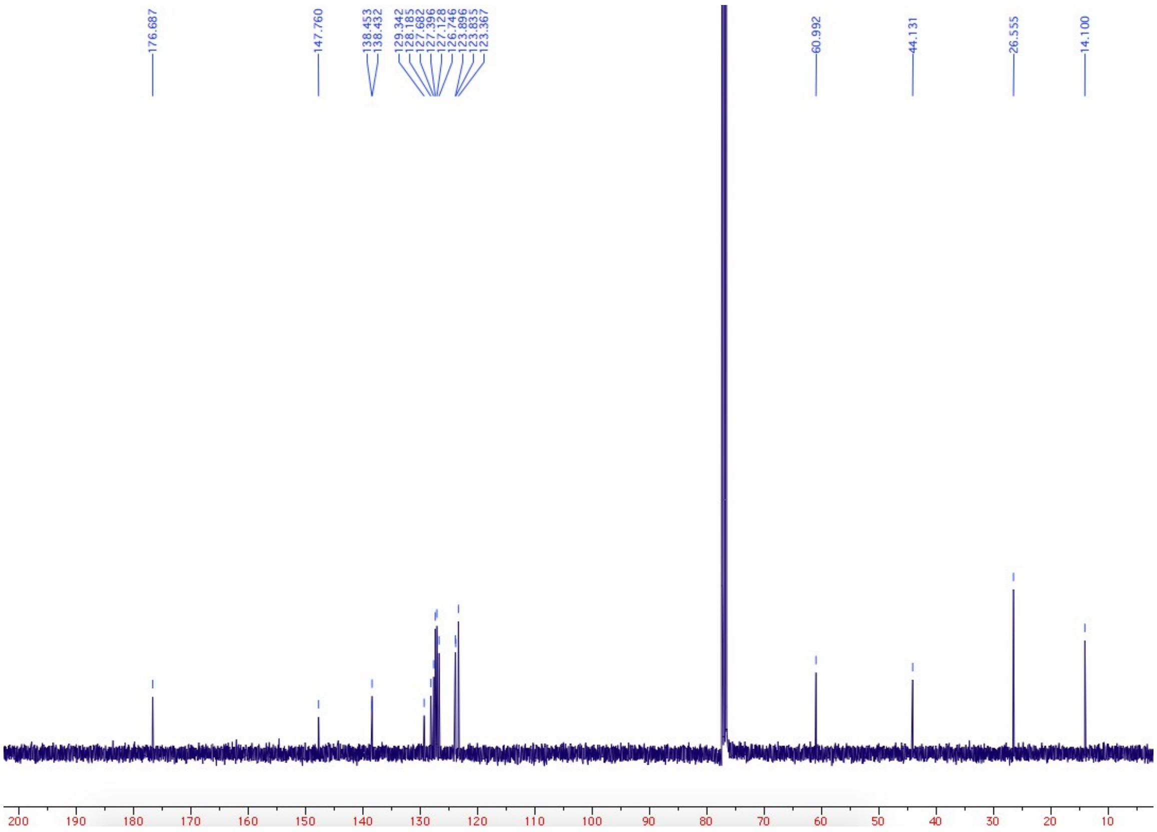

Polymer 21
^{1}H NMR ($CDCl_3$, 400 MHz)

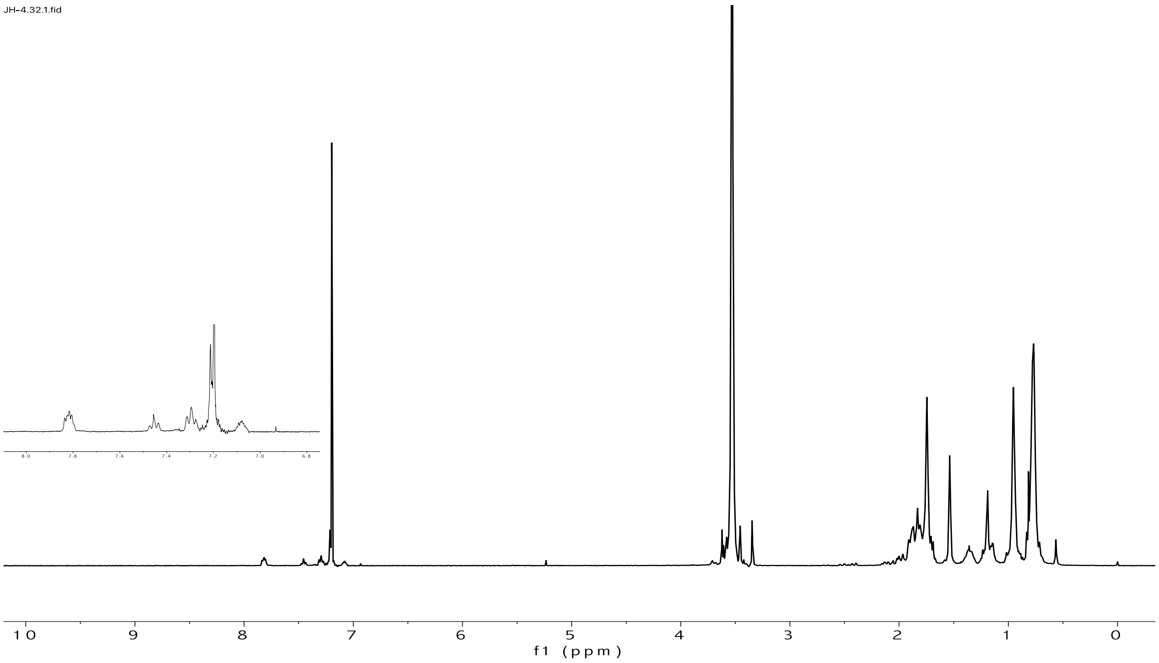

Polymer 22
^{1}H NMR ($CDCl_3$, 400 MHz)

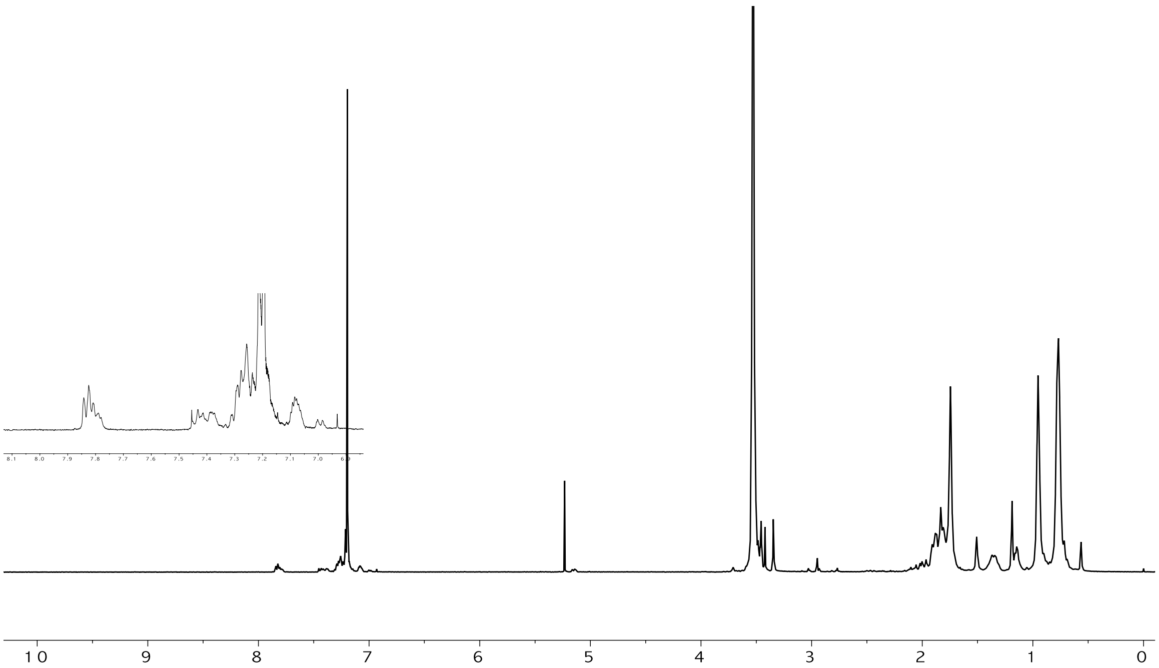

Polymer 23
^{1}H NMR ($CDCl_3$, 400 MHz)

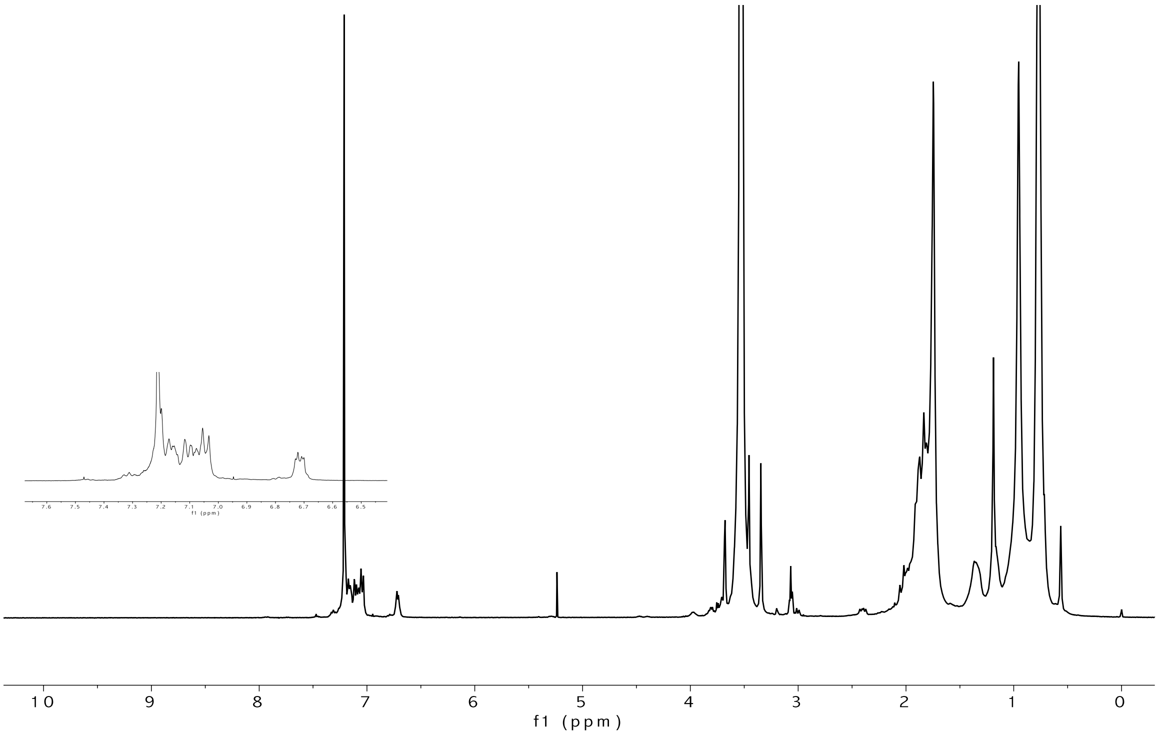

Polymer 24
^{1}H NMR ($CDCl_3$, 400 MHz)

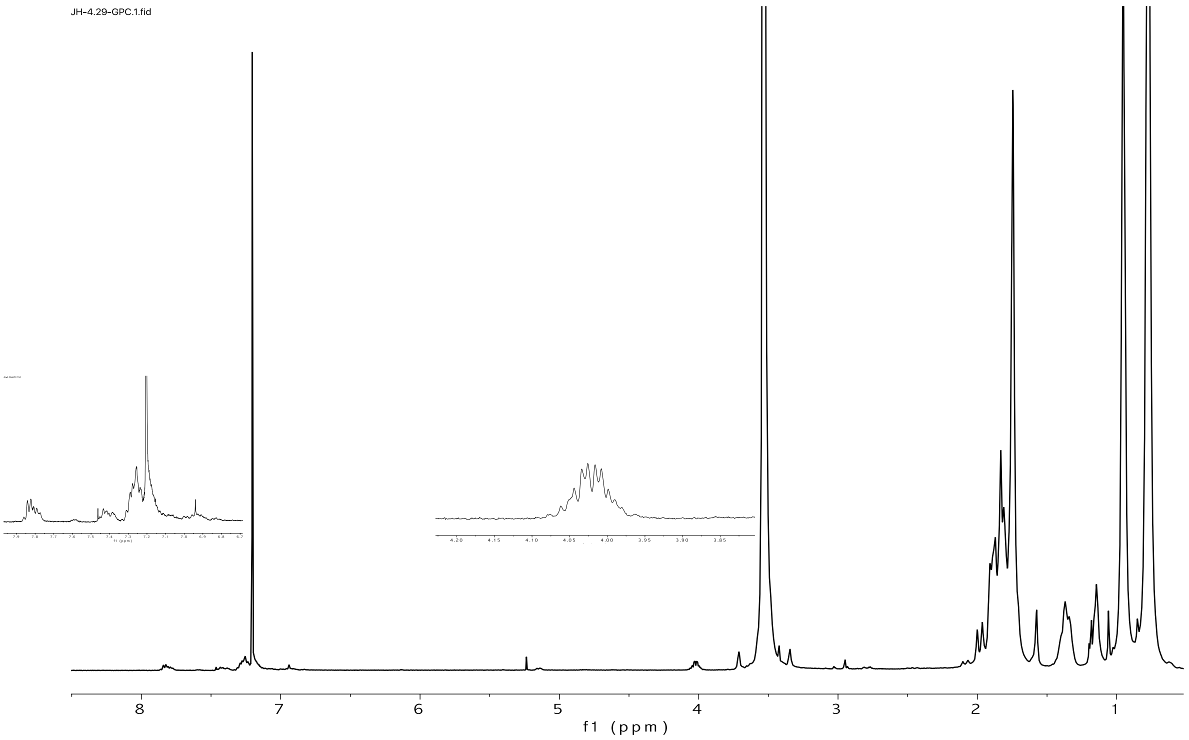

Polymer 25
^{1}H NMR ($CDCl_3$, 400 MHz)

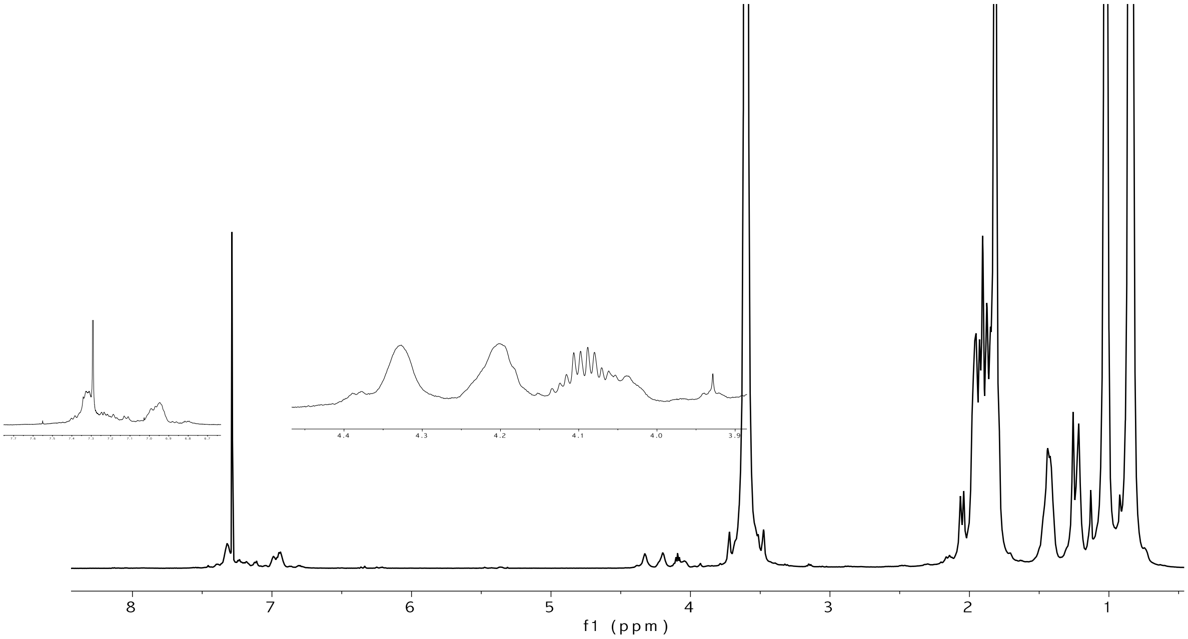

S2
^{1}H NMR ($CDCl_3$, 400 MHz)

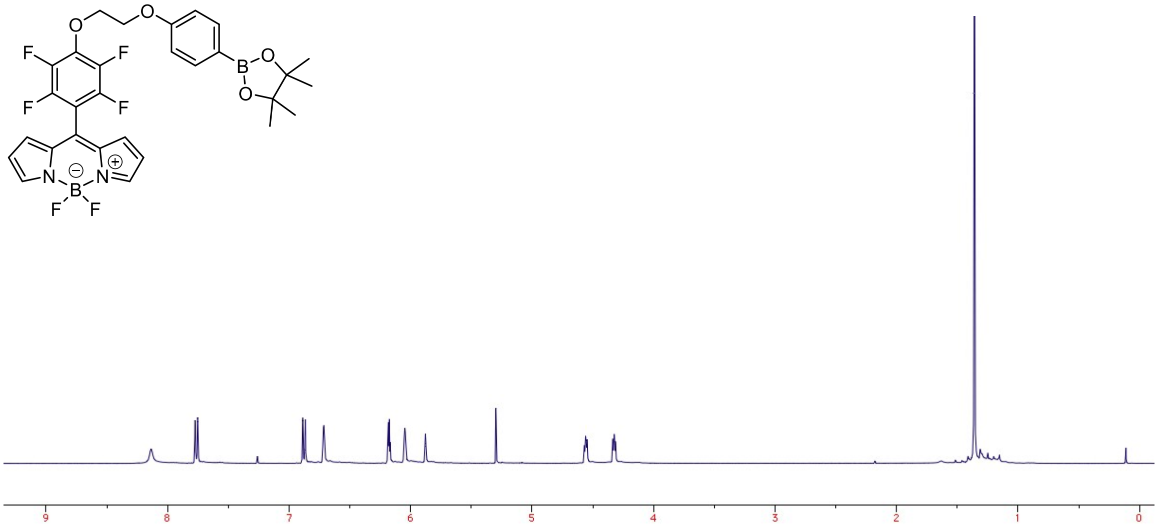

^{13}C NMR ($CDCl_3$, 100 MHz)

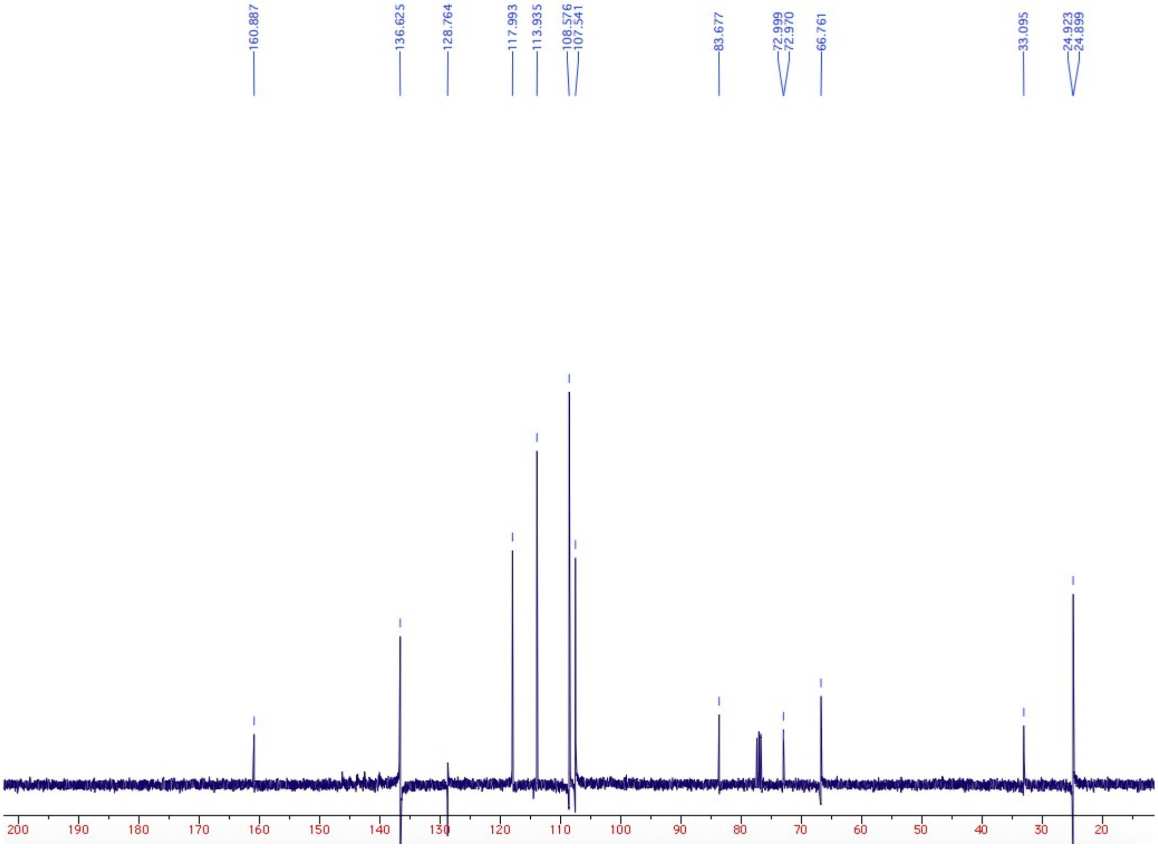

S3

^{1}H NMR ($CDCl_3$, 400 MHz)

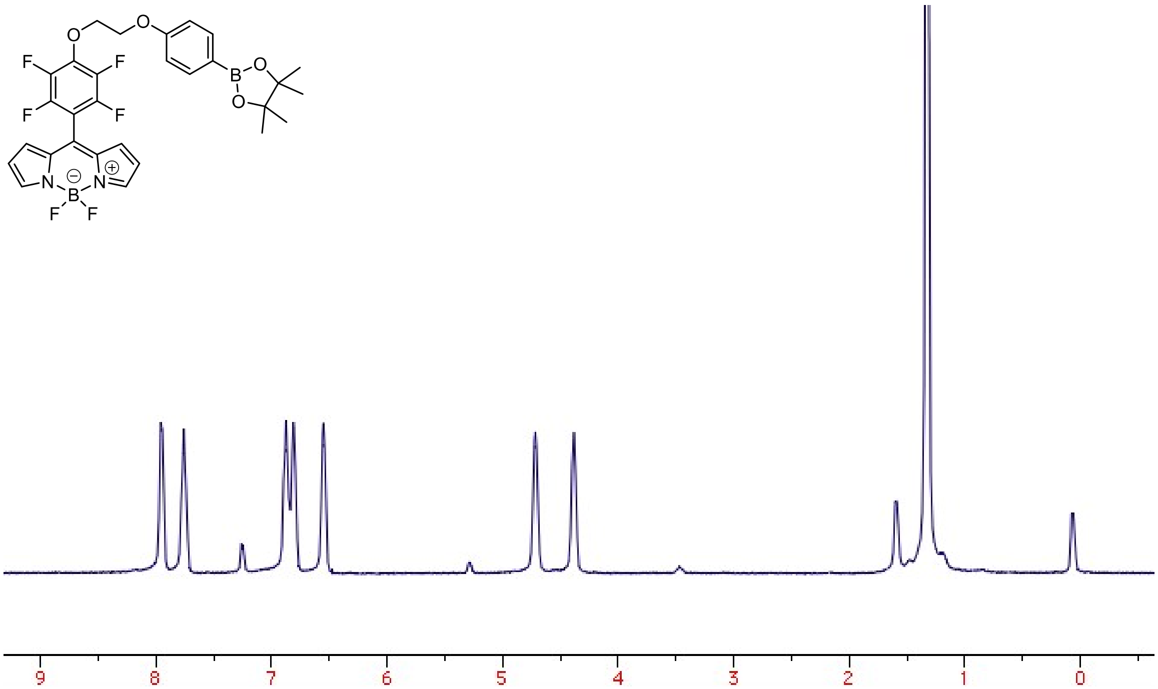

^{13}C NMR ($CDCl_3$, 100 MHz)

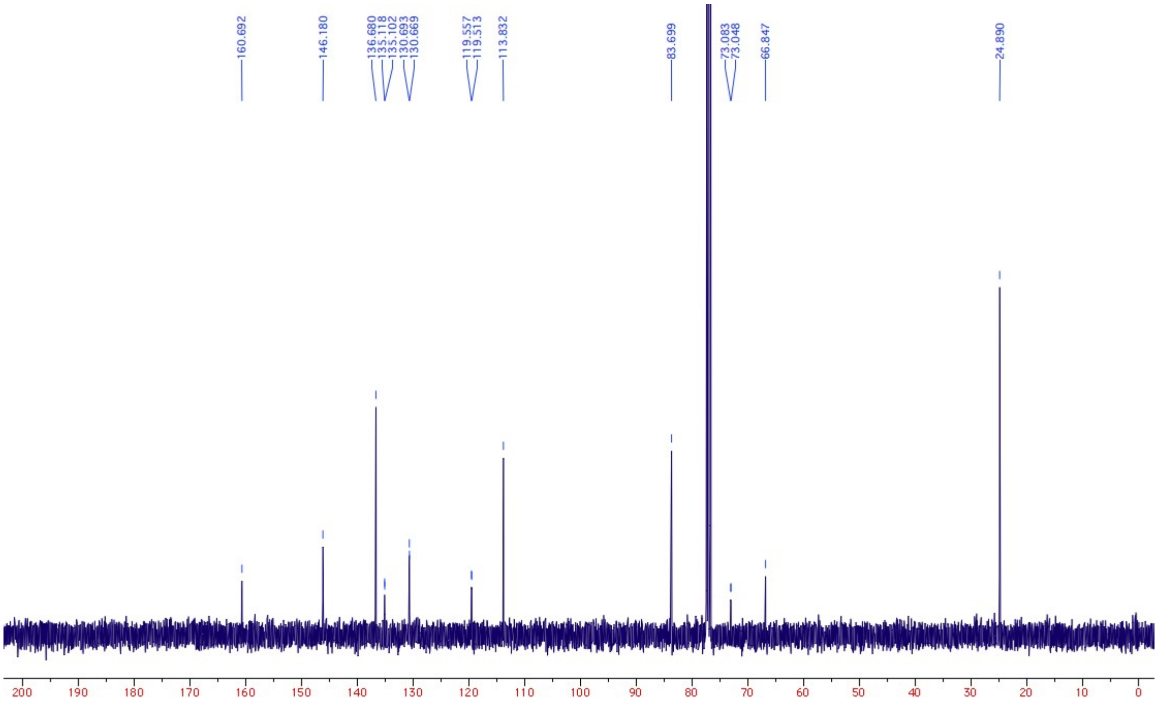

S4

^{1}H NMR (CDCl$_3$, 400 MHz)

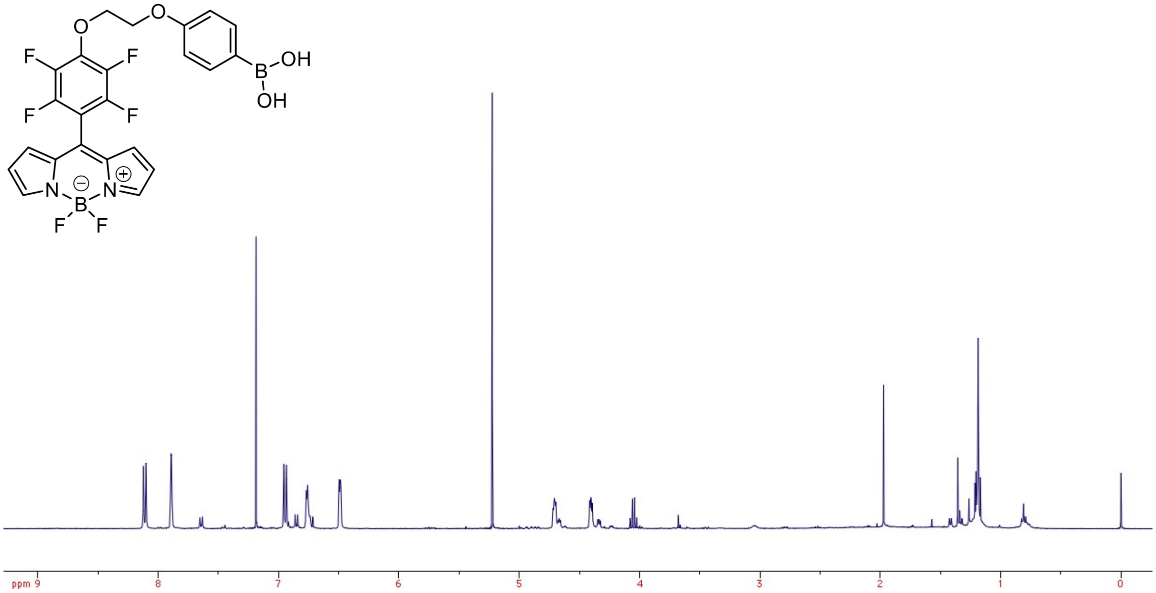

^{13}C NMR (CDCl$_3$, 100 MHz)

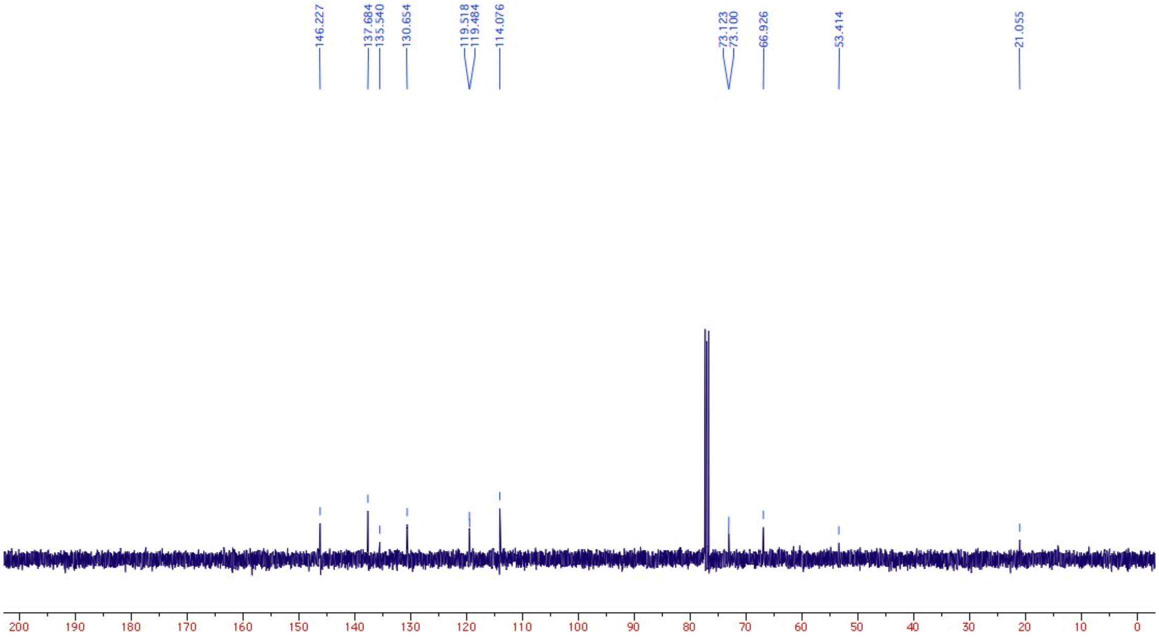

S5

^{1}H NMR ($CDCl_3$, 400 MHz)

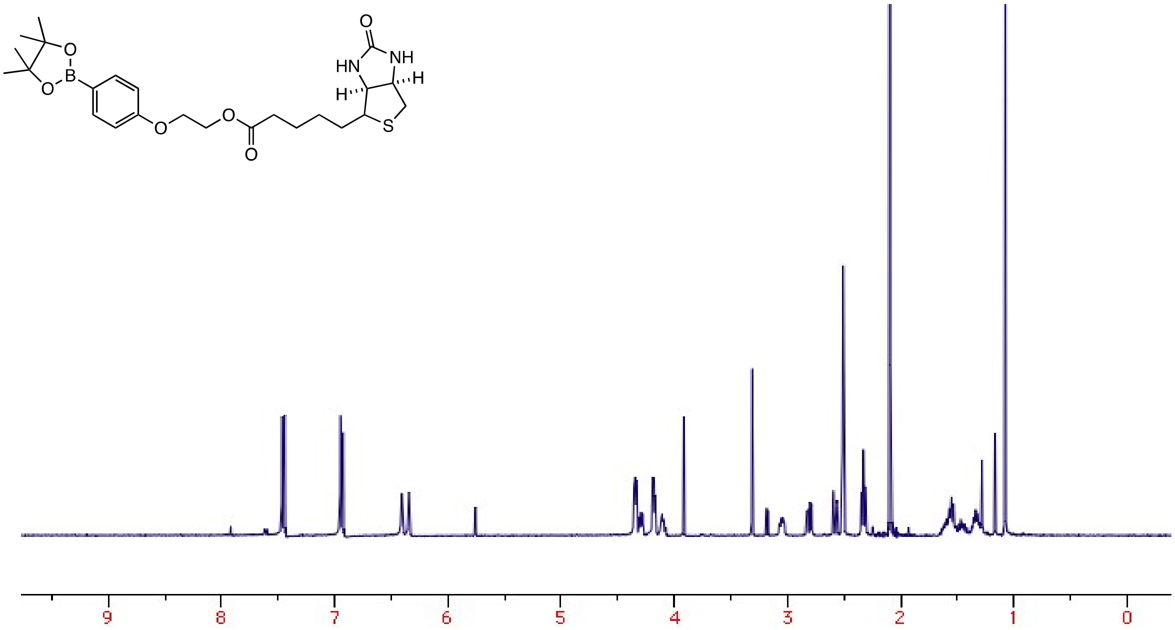

^{13}C NMR ($CDCl_3$, 100 MHz)

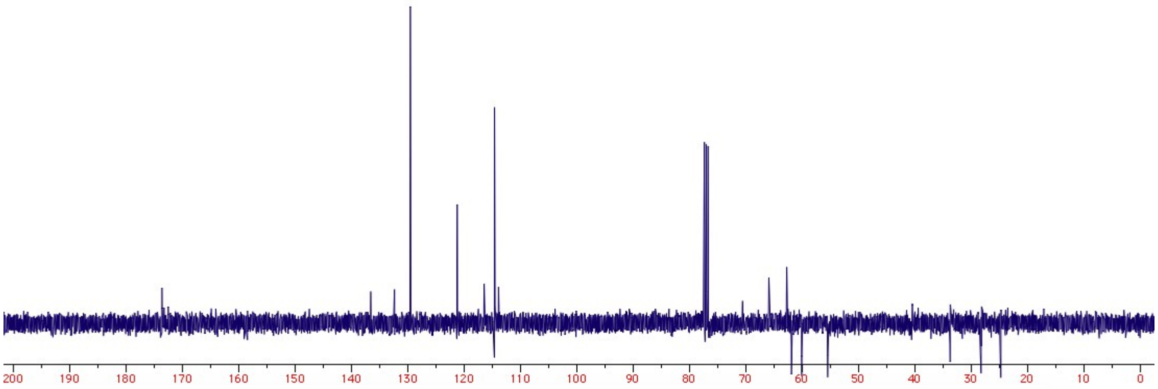

S6

^{1}H NMR (DMSO-d_6, 600 MHz)

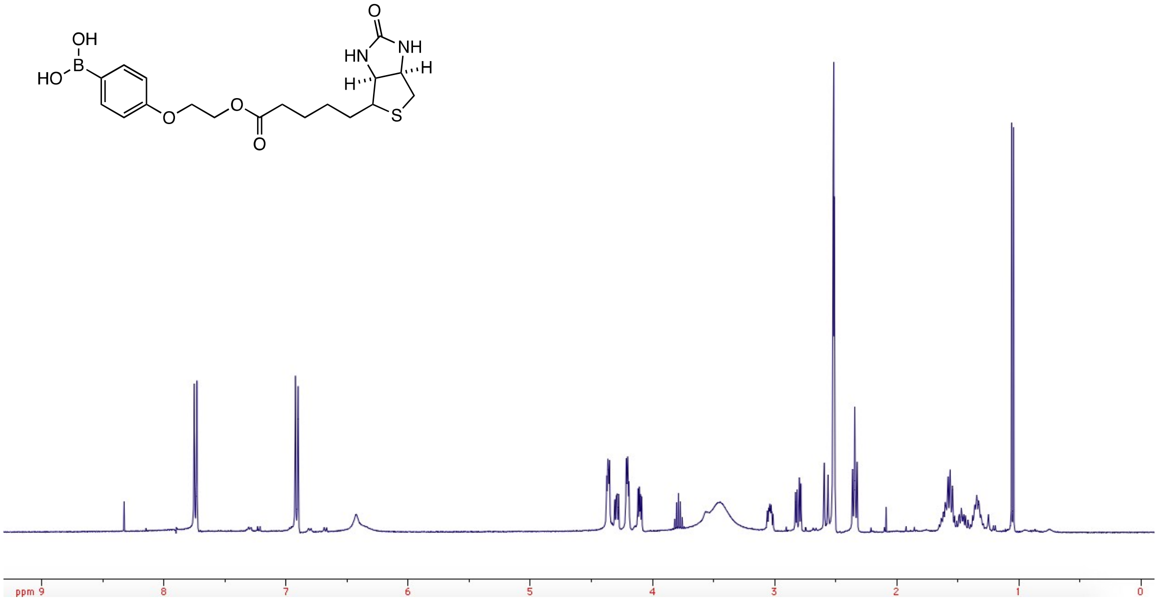

^{13}C NMR (DMSO-d_6, 125 MHz)

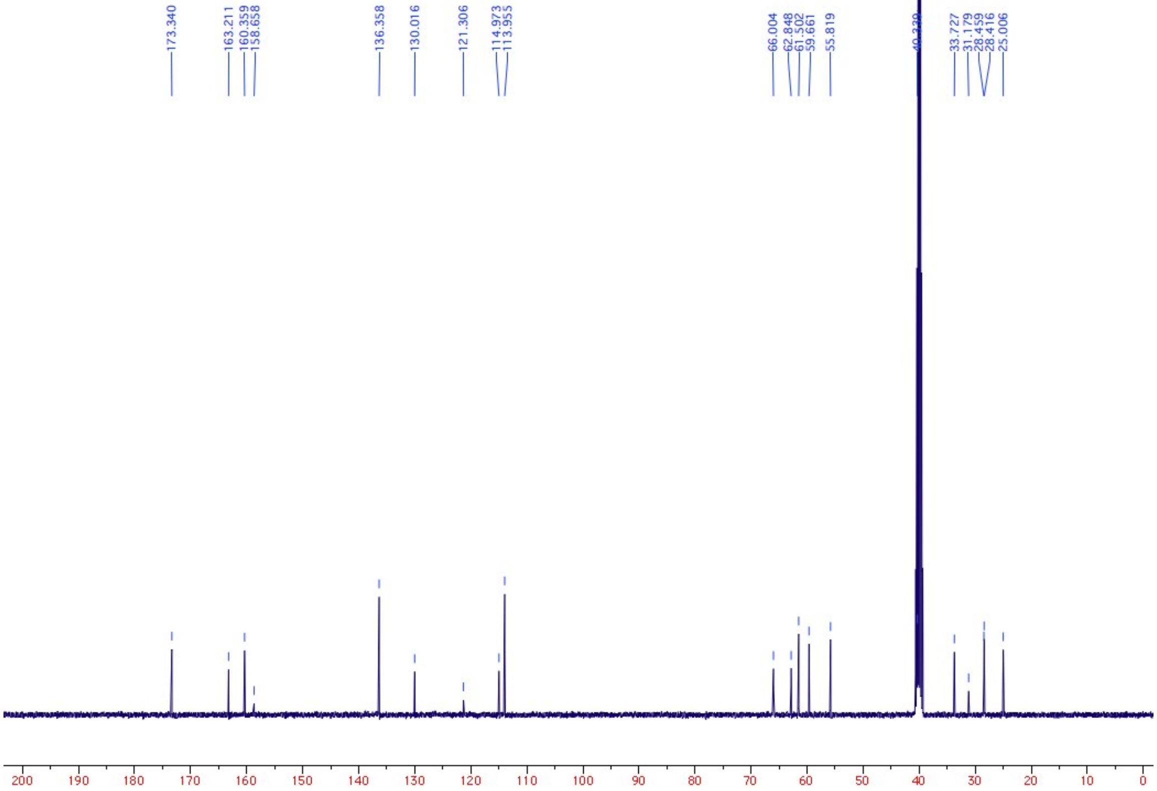

Polymer 26
^{1}H NMR ($CDCl_3$, 400 MHz)

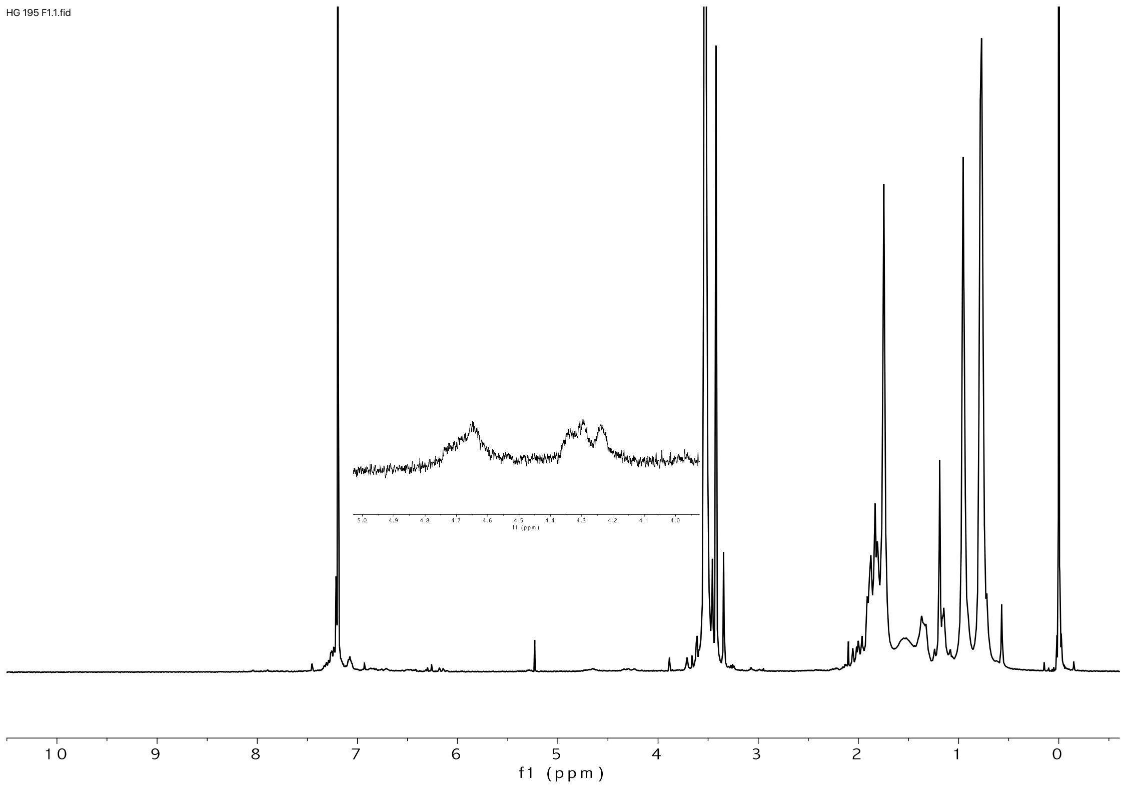

Polymer 27
^{1}H NMR ($CDCl_3$, 400 MHz)

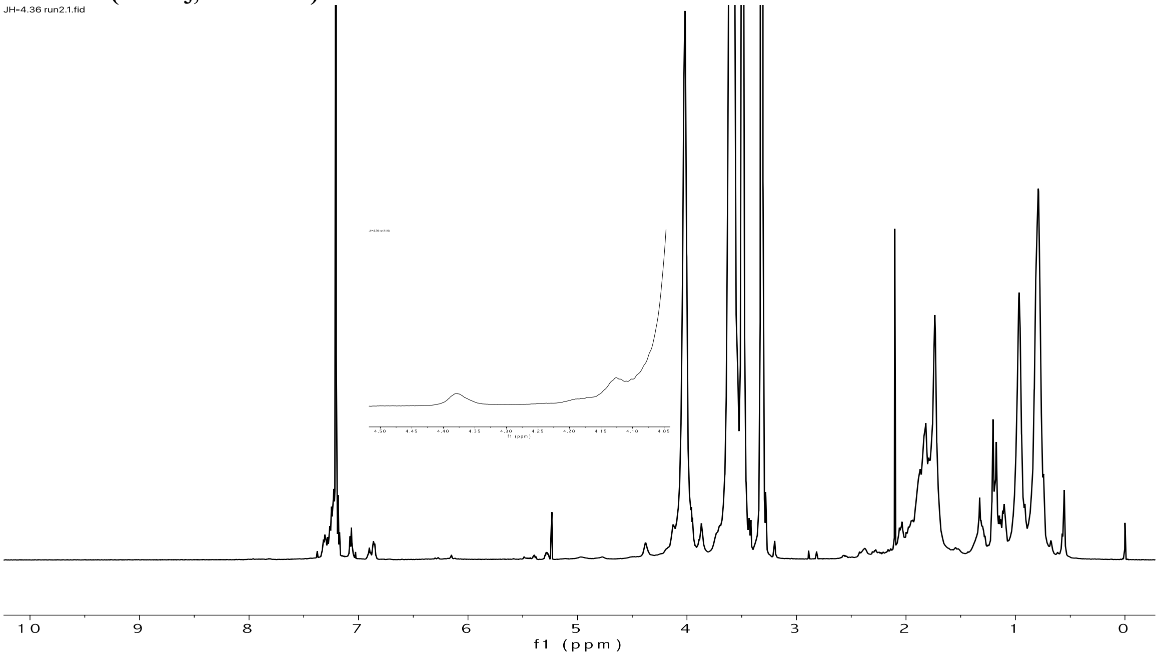

References

1) Grover, G. N.; Alconcel, S. N. S.; Matsumoto, N. M.; Maynard, Heather D. *Macromolecules* 2009, **20***,* 7657-7663.
2) Sinnwell, S.; Synatschke, C. V.; Junkers, T.; Stenzel, M. H.; Barner-Kowollik, C. *Macromolecules,* 2008, **21**, 7904-7912.
3) Peng, L; Li, Y; Li, Y.; Wang, W.; Pang, H.; Yin, G. *ACS Catal.* 2018**, 8**, 2155-5435.
4) Littler, B. J.; Miller, M. A.; Hung, C.-H.; Wagner, R. W.; O'Shea, O. F.; Boyle, P. D.; Lindsey, J. S. *J. Org. Chem.* 1999, **64**, 1391–1396.
